# iBBiG: iterative binary bi-clustering of gene sets

**DOI:** 10.1093/bioinformatics/bts438

**Published:** 2012-07-12

**Authors:** Daniel Gusenleitner, Eleanor A. Howe, Stefan Bentink, John Quackenbush, Aedín C. Culhane

**Affiliations:** ^1^Department of Biostatistics and Computational Biology, Dana-Farber Cancer Institute, Boston, MA, USA, ^2^Department of Statistics, University of Oxford, Oxford, UK, ^3^Department of Biostatistics, Harvard School of Public Health, Boston, MA, USA and ^4^Department of Cancer Biology, Dana-Farber Cancer Institute, Boston, MA, USA

## Abstract

**Motivation:** Meta-analysis of genomics data seeks to identify genes associated with a biological phenotype across multiple datasets; however, merging data from different platforms by their features (genes) is challenging. Meta-analysis using functionally or biologically characterized gene sets simplifies data integration is biologically intuitive and is seen as having great potential, but is an emerging field with few established statistical methods.

**Results:** We transform gene expression profiles into binary gene set profiles by discretizing results of gene set enrichment analyses and apply a new iterative bi-clustering algorithm (iBBiG) to identify groups of gene sets that are coordinately associated with groups of phenotypes across multiple studies. iBBiG is optimized for meta-analysis of large numbers of diverse genomics data that may have unmatched samples. It does not require prior knowledge of the number or size of clusters. When applied to simulated data, it outperforms commonly used clustering methods, discovers overlapping clusters of diverse sizes and is robust in the presence of noise. We apply it to meta-analysis of breast cancer studies, where iBBiG extracted novel gene set—phenotype association that predicted tumor metastases within tumor subtypes.

**Availability:** Implemented in the Bioconductor package iBBiG

**Contact:** aedin@jimmy.harvard.edu

## 1 INTRODUCTION

Genomic studies have generated vast quantities of data, including gene expression, copy number variation and single-nucleotide polymorphisms. Tens of thousands of gene expression profiling experiments are stored in public repositories (Barrett *et al.*, 2009; Parkinson *et al.*, 2009) and are increasingly studied in aggregate. The aim of these studies is typically to discover a set of genes that participate in a pathway and are robustly predictive of a biological phenotype in a meta-analysis of multiple studies.

Gene set analysis (GSA) was developed to identify such gene sets whose expression distinguishes biological conditions, even if single-gene analysis fails to find significant associations with the phenotype. The method takes advantage of *a priori* defined gene sets published in gene set databases (Gene Ontology, KEGG and MSigDB) or resulting from differential expression studies (MSigDB and GeneSigDB) (Culhane *et al.*, 2009, 2012; Subramanian *et al.*, 2005). GSA has been successfully applied to the analysis of microarray experiments (Goeman and Buehlmann, 2007; Mootha *et al.*, 2003) and has been extended beyond transcriptomics to other areas, such as analysis of genome-wide association studies (Cantor *et al.*, 2010; Wu *et al.*, 2008). When compared to traditional single-gene analysis that ranks differential gene expression between two conditions, GSA produced more consistent biological results across studies even when some genes in a gene set were absent or poorly represented in a dataset (Fan *et al.*, 2011). Although GSA naturally extends to integrated meta-analysis, surprisingly few studies have applied meta-GSA to integration of multiple datasets (Segal *et al.*, 2004; Montaner and Dopazo, 2010). Most meta-GSA approaches are designed for the limited case, where datasets have matched samples or features (Montaner and Dopazo, 2010; Tyekucheva *et al.*, 2011). These either create a merged dataset of features (genes) common to all datasets and perform a GSA or apply GSA on each individual datasets and then combine the resulting GSA statistics or *P*-values to produce a ranked list of gene sets (Montaner and Dopazo, 2010; Shen and Tseng, 2010; Tyekucheva *et al.*, 2011).

While a single ranked list of gene sets may capture the biological complexity of a simple cellular system, it is insufficient when applied to the study of complex disease or meta-analysis of large numbers of studies where different pathways are active in different subsets of samples. These limitations can be addressed using bi-clustering, a simultaneous similarity-based clustering of features and conditions, resulting in modules; subsets of features that exhibit consistent patterns over subsets of conditions (Cheng and Church, 2000). Numerous bi-clustering approaches have been applied to continuous (Cheng and Church, 2000; Hochreiter *et al.*, 2010; Huttenhower *et al.*, 2009) and discretized gene expression profiles (Prelic, 2005); however, these methods have not been applied in meta-GSA.

We introduce **i**terative **B**inary **Bi**-clustering of **G**ene sets (iBBiG), a new bi-clustering algorithm to perform meta-GSA that addresses the shortcomings of ‘ranked list’ meta-GSA approaches. It scales well when applied to hundreds of datasets is tolerant to noise characteristic of genomics data and when applied on simulated data, outperforms clustering and bi-clustering methods including hierarchical and *k*-means clustering, FABIA (Hochreiter *et al.*, 2010), COALESCE (Huttenhower *et al.*, 2009) and Bimax (Prelic, 2005). To perform meta-GSA, we first transform ‘noisy’ gene expression profiles to simple binary gene set profiles, which simplifies the conjoint study of different studies as it negates the need to match probes across platforms. We then apply iBBiG to extract clusters or ‘modules’ of groups of phenotypes whose gene expression profiles are enriched in similar gene sets (Supplementary Fig. 1). An attractive feature of iBBiG is that it does not require prior knowledge or limit the number or size of clusters, a non-trivial requirement in cluster analysis of most large biological datasets. The results of iBBiG are easy to parse; iBBiG modules are ranked by an information score, and within each module, gene sets are ranked by a fitness score that measures its weight in the module. It uses a genetic algorithm to maximize the size and entropy of each bi-cluster producing a small number of bi-clusters whose functional and phenotypic associations can be easily interpreted; eight modules were associated with known breast cancer clinical covariates in meta-GSA of 21 breast cancer gene expression datasets, and we detected 13 modules prognostic both within and across breast cancer molecular subtypes using a cluster discovery approach, which ignored *a priori* sample knowledge.

## 2 MATERIALS AND METHODS

We tested the ability of iBBiG to discover bi-clusters in matrices of real and simulated data. Real gene expression data were transformed to gene set profiles using two different GSA approaches (i) single sample and (ii) pairwise test which were computed using the Bioconductor packages gene set variation analysis (GSVA) and GSEAlm (Oron *et al.*, 2008), respectively. GSVA ranks gene sets within each individual gene expression profile to produce a gene set by sample matrix. GSEAlm tests for enrichment of gene sets in a ranked list of genes that are differential expressed between two conditions or clinical covariates (e.g. Grade 1 versus Grade 3) resulting in a gene set by pairwise test matrix. Resulting GSA *P*-values are discretized generating a spare binary matrix. Columns contain ‘phenotypes’ defined by single sample or a pairwise test GSA results, in which 1 is a significant association (*P* < 0.05) between a gene set and phenotype and 0 represents a lack of association.

### 2.1 Datasets

#### 2.1.1 Single sample GSA data

Normalized gene expression of primary breast tumors was download from GEO (GSE20685, *n* = 327) (Kao *et al.*, 2011) or imported from the Bioconductor data packages breastCancerNKI (*n* = 337) (van't Veer *et al.*, 2002), and breastCancerVDX (*n* = 344) (Minn *et al.*, 2007). Data were obtained on Affymetrix U133 Plus2, Agilent and Affymetrix U133a GeneChip arrays; each contained different numbers of features. GSVA was applied using gene sets from C2 subset of MSigDB v3.0 to transform gene expression data into gene set *x* sample matrices. No prior knowledge or covariates were used in GSVA. The resulting values were discretized (*es.os*≤±0.3) to produce an association matrix of sparse (11.6%, *n* = 1) binary data of 1008 columns (breast cancer samples) and 5098 rows (up and down-regulated results for 2459 gene sets). iBBiG was applied with default parameters with nModules = 20.

#### 2.1.2 GSEAlm data

To discover modules associated with known breast cancer clinical covariates, 21 normalized breast cancer gene expression datasets (3875 gene expression profiles, see Supplementary Table S1) annotated with 107 clinical covariates were obtained from the GeneChip Ontology Database (Liu *et al.*, 2011). Clinical covariates included tumor grade, stage, age or hormone status. GSEAlm (Oron *et al.*, 2008) was applied to all pairwise combinations of each covariate. For example, if the covariate grade has levels 1, 2 and 3, all possible pairwise combinations would result in six phenotypes (Grades 1v2,1v3,2v1,2v3,3v1 and 3v2), therefore pairwise tests of the 107 covariates resulted in 448 phenotypes. GSEAlm was performed independently and we did not merge studies at the probe (gene), sample or phenotype level. Gene sets from the C2 and C5 subsets of MSigDB v2.0 (*n* = 2293) (Subramanian *et al.*, 2005) and GeneSigDB v1.0 (*n* = 560) (Culhane *et al.*, 2009) were used. Resulting *P*-values were corrected for multiple testing using the false discovery rate (Benjamini and Hochberg, 1995) and discretized (*P* < 0.05), to produce an association matrix of sparse binary data with 448 columns (pairwise tests) and 2853 rows (gene sets). iBBiG was applied to detected 50 modules. Examination of the fitness score and module size plots (Supplementary Fig. 24) identified eight modules that had a fitness score of over 1000 and a minimum of five pairwise tests.

#### 2.1.3 Simulated data

**Fig. 1:**
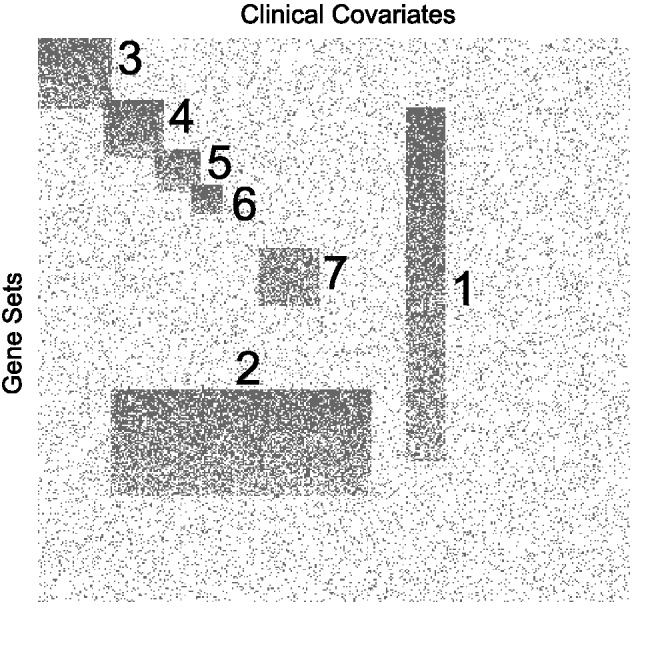
Data were simulated to represent discretized *P*-values from GSA of 400 pairwise tests and 400 gene sets. Associations are shown in gray, whereas non-associations are in white. The dataset has 10% background noise and contains seven overlapping clusters of gene set modules. These include overlaps of pairwise tests (columns), overlaps of gene sets (rows) and overlaps in both dimensions. There were different signal strength gradients with each module M1 (90–40%), M2 (80–50%), M3 (80–40%), M4 (90–50%), M5 (80–40%), M6 (90–40%) and M7 (60–50%)

using observations of GSA results of real data, we simulated a dataset of 400 pairwise tests by 400 gene sets in which we placed seven modules (M1–M7; Fig. 1). These were introduced by assigning associations (value of 1) to column and row pairs. To replicate the expected properties of real data, modules were created such that they partially overlap in columns, in rows and in both directions; M1 gene sets overlapped with all modules except M3. M2 overlapped pairwise tests with modules M4–7. Artificial modules had highly variable sizes and included ‘wide’ modules driven by a large number of pairwise tests with few gene sets and ‘tall’ modules like M1 which contained 25 pairwise tests with a large number of gene sets (*n* = 250). This latter module (with many gene sets) might represent a complex, well-characterized biological process such as proliferation. Random background noise (at 10%), which is characteristically observed in genomics data, was also added. In real data the signal strength will vary both between and within modules. Therefore, to simulate variance among modules, random noise (replacing values of 1 with 0) was imposed to produce modules with different signal strengths (Fig. 1). Within a module, we expect to see few strong signals (gene sets associated with all pairwise tests) and many weaker signals. Therefore, within each module, a noise gradient was also applied so that the first gene sets had a greater number of associations (Fig. 1). This within-noise gradient ranged from 10 to 60% and varied between modules. The size, signal strength and signal gradient of each module are provided in Table 1. Overlaps are visualized in Figure 1. R code to generate this data (using the function makeArtificial available in our Bioconductor package iBBiG) is provided in Supplementary Materials. iBBiG was also applied to the 21 real datasets in which the sample labels were permuted by random shuffle, but only recovered small modules (2 and 3 pairwise tests) with low or negative weighted scores (data not shown).

**Table 1:** Module nomenclature (M1–M7) is the same as that used in Figure 1

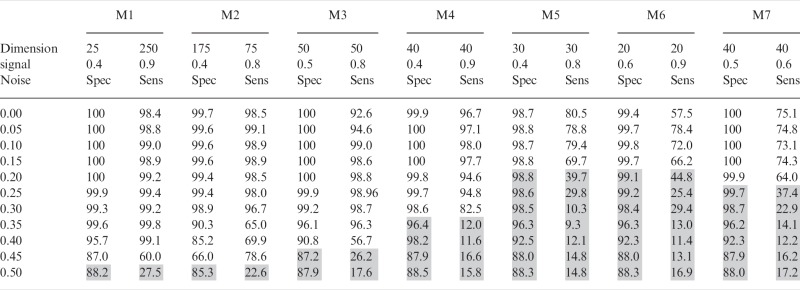

The first row indicates the dimension (number of columns and number of rows) of each module in the simulated dataset. The signal strength gradient within each module from the strongest to weakest signal-to-noise ratio is provided in the second row. For example M1 has 25 pairwise tests (columns) and 250 gene sets (rows) and a signal-to-noise is 0.9 in the first few rows which decreases to 0.4 as the number of gene sets reaches 250. The remainder of the table shows the impact of increasing background noise (between 0 and 50%) on the mean specificity and sensitivity of 100 analyses (alpha = 0.3, selection pressure = 1.2, population size = 100, mutation rate = 0.08, success ratio = 0.6). Results in which either sensitivity or specificity drop below 50% are highlighted with a grey background.

### 2.2 Methods

The iBBiG algorithm identifies bi-clusters (or modules) in a matrix of binary data and consists of three main components (i) a module fitness score (ii) a heuristic search algorithm to identify and grow modules in a high dimensional search space and (iii) an iterative extraction method to mask the signal of modules that have already been discovered.

#### 2.2.1 Fitness score

The module fitness score measures both module size and homogeneity. A module yields a high fitness score when a large group of phenotypes are associated with the same features (gene sets). We use the term phenotype to indicate a binary vector of discretized *P*-values resulting from either a pairwise test (GSEAlm) or single sample (GSVA) GSA. Module homogeneity is evaluated using Shannon's Entropy (Shannon, 1948), a standard approach to measure homogeneity, often used in cluster analysis Jenssen *et al.* (2003) and Li *et al.* (2004). An asymmetrical score was used; associations are considered and non-associations that can result from multiple technical causes are ignored. Assume a binary matrix *M*, with *m* columns (phenotypes) and *n* rows (features or gene sets), in which an element *m*_*ij*_ {1,0} represents an association between phenotype *j* and gene set *i*. Given a module *K* with *k* phenotypes, where 2 ≤ *k* ≤ *m*, the probability of an association between a gene set *i* and the phenotypes in module *K* is
(1)
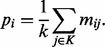



Subsequently, the entropy *H*_*i*_ of gene set *i* and phenotypes in *K* can be calculated
(2)
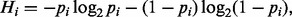

where 0.log_2_ 0 = 0 (Shannon, 1948). *H*_*i*_ has the range 0 ≤ *H*_*i*_ ≤ 1, where *H*_*i*_=0 when a gene set is associated (*p*_*i*_=1) or not associated (*p*_*i*_=0), with all *k* phenotypes. *H*_*i*_=1 when associations are random *p*_*i*_ = 0.5.

The score *S*_*i*_ of gene set *i* in module *K* is weighted. The weight matrix *W* has the same dimension as matrix *M* and equals *M* on the first iteration (when finding the first module), but for subsequent iterations an element *w*_*ij*_ of *W* is modified if *m*_*ij*_ was included in a module. The weight *W*_*ik*_ for gene set *i* in a module *K* is given as
(3)
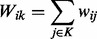

(4)
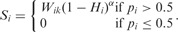



The parameter α (range 0 < α < 1.0) is a weighting factor that balances module homogeneity and module size (number of phenotypes versus number of genesets). Clearly one could have a large module with low homogeneity and vice versa. Consequently, α balances the tradeoff between specificity and sensitivity (Supplemental Fig. 3). The optimal default value (described below) was determined to be α = 0.3 (Supplementary Table 2). Supplemental Figure 2 depicts the behavior of the entropy-based score for a single gene set. A score for an entire module *S* is calculated by summing up the *n* gene set scores *S*_*i*_, which can be optimized with the addition or omission of phenotypes. A beneficial side effect of this approach is the weighting of gene sets (Supplementary Figs. 4 and 5). The weight indicates the importance of a gene set to a module, relative to other gene sets in that module. This ranking allows comparison of the relative importance of biological processes represented by those gene sets.

#### 2.2.2 Genetic algorithm

Due to the high dimensionality of the association matrix, it is not feasible to search the solution space of all possible modules exhaustively; hence we use a genetic algorithm. Genetic algorithms (GAs) are a class of heuristic search algorithms and a particular form of optimization methods based on evolutionary concepts. They use natural selection, recombination and sexual reproduction in order to find heuristic solutions for optimization problems. In the GA implemented in iBBiG, a module (individual) is represented by a binary vector with length equal to the number of phenotypes, where 1 indicates membership of a phenotype within a module. A population of such individuals is initialized by randomly selecting two phenotypes. The fitness of each individual is evaluated using the entropy-based gene set score. High-fitness individuals are chosen as parents to create the next generation of solutions, using a linear ranking selection operator (Chakraborty and Chakraborty, 1997). A single-point crossover operator is used for recombination and a bit flip operator for the mutation operator. An offspring selection operator is used to introduce self adaptive selection pressure (Affenzeller and Wagner, 2005). The algorithm produces generations of solutions, until the highest fitness score in the population stagnates for a specified number of generations.

#### 2.2.3 Iterative module extraction

The bi-clustering approach described so far is able to find one module at a time. In order to find all possible clusters, an iterative approach similar to that described by Huttenhower *et al.* (2009) was applied. After finding a module *K*, the weights of associations (*W*_*ik*_) between gene set *i* and phenotypes *k* in module *K* are subtracted from the weighting matrix *W* and the bi-clustering is applied again,

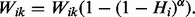

Few gene sets have associations with all *k* phenotypes (*H*_*i*_ = 0). Only the portion of the signal used to calculate the fitness score is removed, as a result a residual association signal *H*_*i*_ > 0 remains in the weight matrix. Residual information not used in modules up to the current iteration is available for subsequent iterations, ensuring iBBiG can find true overlapping clusters.

#### 2.2.4 Optimization of iBBiG input parameters

The optimal range for each parameter of the GA was tested in 100 runs on simulated data (Supplementary Tables S3–S7). The only parameter that had an impact on the performance was the α parameter that regulates the weighting between the homogeneity and module size (number of phenotypes) (Supplementary Fig. S3). Experiments in which the α parameter was varied 0.1 < α < 0.9) (Supplementary Table S2) show that the clustering on the simulated datasets performs optimally (specificity 99.7% and sensitivity 90.5%) with a value of α = 0.3. Most other parameters had little effect. Higher population size *P*, which is required to establish the necessary amount of genetic diversity of solutions, shows only marginal differences in sensitivity and specificity (Supplementary Table S3). The same is true for the mutation rate *MR* (Supplementary Table S4), the selection pressure for parent selection *SP* (Supplementary Table S5) and the success ratio *SR* that determines how many children have to outperform at least one of their parents (Supplementary Table S6). The optimal default settings were determined to be an α of 0.3, a population size of 100 individuals, a mutation rate of 0.08, a success ratio of 0.6, a selection pressure of 1.2 and a stop criterion of 50 iterations of stagnation.

#### 2.2.5 Comparison to other clustering methods

*K*-means clustering was performed using the ‘stats’ library in R with the parameter *K* = 7. Hierarchical clustering was applied to an asymmetrical binary distance matrix using Ward's minimum variance method in both dimensions. Bi-clustering methods: δ clustering (Cheng and Church, 2000), xMOTIF (Murali and Kasif, 2003), Bimax (Prelic, 2005), Plaid (Turner *et al.*, 2005) and SPEC (Kluger, 2003) were applied using default parameters using the R packages ‘biclust’ and ‘stats’, respectively. Over 130 runs of Bimax were performed to optimize module size parameters minr (2–26) and minc (2–20) that define module row and column size. FABIA (Hochreiter *et al.*, 2010) was applied using the R library ‘fabia’ to find eight clusters (*p* = 8) in 1000 cycles (*cyc* = 1000) with different spareness loading (α = 0.1, 0.2 or 0.3). COALESCE (Huttenhower *et al.*, 2009) available in the Sleipnir package was performed using default parameters for both initial cluster discovery and post-processing (COALESCE -j). Each method detected different clusters, and results show the predicted clusters with maximum pairwise Jaccard similarity index (JI) to each of the ‘true’ modules M1–M7. In Supplementary Tables S10–16, JI was calculated over phenotype membership, ignoring the ranking of the gene sets. To assess iBBiG's ability to assign gene sets to clusters (Supplementary Tables S17), gene set scores for each predicted module were subjected to Gaussian mixture modeling using the R function ‘mclust’ to discriminate gene sets with high scores from the background null distribution.

#### 2.2.6 Implementation

Documentation and code are available in the Bioconductor R package iBBiG. The core functions of the genetic algorithm were implemented in C to speed computation.

## 3 RESULTS

### 3.1 Robustness of iBBiG in the presence of increasing noise

iBBiG maintained high performance predicting the strongest signals (M1 > M2 > M3) in the presence of up to 45% noise when background noise was increased in 5% increments up to 50% (Table 1). The weaker signal modules (M4–M7) show a high level of specificity but a decline in sensitivity starting at 20% noise. Increasing the α parameter (from the default of 0.3 to 0.5) increased sensitivity with a trade-off of decreased specificity. The algorithm detected M4 to M7 at 25% noise, with an α of 0.5 (Supplementary Table S7). In addition, we evaluated the stability of gene set scores over 100 runs (Supplemental Fig. S4). Within each module, gene sets are ranked by a score that indicates the number of phenotypes in which a gene set is differentially regulated. We observed that gene sets with high scores showed low variation among runs (Supplemental Fig. S4).

### 3.2 Performance of global and bi-clustering methods on simulated meta-GSA data

**Fig. 2:**
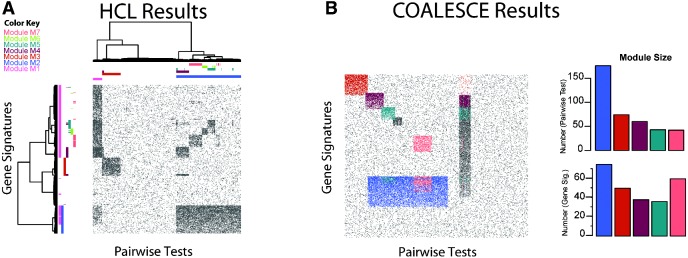
Neither **(A)** hierarchical clustering or **(B)** COALESCE could detect all seven modules (M1–M7) in cluster analysis of the simulated dataset. True modules are shown in dark gray, and detected modules are colored M1–M7 as indicated. **(A)** Rows and columns are ordered by hierarchical cluster analysis (Wards minimum variance). Plots of the JI, which measured similarity between the true and predicted clusters when the dendrogram was cut to give 3 ≤ *K* ≤ 15, are provided in the Supplement and show that *K* = 8 was optimal, but produced clusters that detected smaller overlapping clusters at the cost of the large clusters (M1 and M2). The larger modules could only be detected when the dendrogram was cut to produce fewer clusters. **(B)** Rows and columns have the same order as Figure 1. COALESCE detected five of the seven modules and had difficulty detecting M1 and M6. Bar plots show the predicted module size (number of phenotypes and gene sets)

Global clustering methods (hierarchical clustering, *k*-means) had difficultly detecting overlapping modules in the simulated dataset as shown in Figure 2A and Supplementary Figures 6A and 7, respectively. Although hierarchical clustering discovers M3 with high specificity and sensitivity, it was unable to detect overlapping clusters; it either identified the large clusters (M1 and M2) or the smaller clusters depending on the height of the dendrogram cut. Supplementary Figure 6B and C shows the maximum JI between predicted and true clusters when dendrogram was cut to give 3–15 clusters. When hierarchical clustering was performed to produce the optimal number of clusters (*K* = 8) with the highest average JI (Figures 6B and C), it was still unable to discriminate M2 and its overlapping clusters M5 and M6 which were all contained within its cluster 3 (Supplementary Table S10).

Most genomics bi-clustering methods are not optimized for binary data as they have been developed for gene expression data with continuous values. Only FABIA, COALESCE and Bimax discovered bi-clusters in the binary simulated data matrix, the others tested (δ-clustering, Plaid, SPEC, xMOTIF) either did not accept a binary matrix as input or failed to find clusters. FABIA performed only moderately well when applied to the artificial dataset (Fig. 8). It tended to discover clusters with large numbers of phenotypes which contained many false positives (Supplementary Fig. 8), for example the first cluster contained almost all phenotypes (358/400) (Supplementary Table S12). Increasing the alpha parameters from default (α =0.1) to 0.2 or 0.3 (Supplemental Tables α =0.2:13, α =0.3: 14) did improve its ability to detect smaller clusters but still produced clusters with high numbers of phenotypes and few gene sets. The mean module specificity and precision of FABIA were 0.71, 0.71, 0.74 and 0.39, 0.38, 0.35 for alpha 0.1, 0.2, 0.3, respectively. COALESCE outperforms most bi-clustering algorithms when applied to gene expression data (Huttenhower *et al.*, 2009), however only detected five of the seven clusters when applied to the simulated data (Fig. 2B, Supplementary Table S11). It was unable to discover the ‘tall’ module M1, which has few phenotypes with a large number of gene sets and the smallest module M6 which has overlaps in both M1 and M2.

Binary inclusion-maximal bi-clustering (Bimax) is arguably one of the most popular binary bi-clustering algorithms, but it is not optimized to tolerate noise in the signal (Prelic, 2005) and produced small clusters with high specificity and poor sensitivity in our simulated dataset (Supplementary Table S16). Bimax requires that the number and size of clusters be specified as input parameters. The simulated data contained modules of different sizes, therefore we tested a large range of minimum row (2 ≤ *minr* ≤ 26) and column size (2 ≤ *minc* ≤ 20), but the maximum JI to M1-M7 ranged from 0.14 (M2) to 0.68 (M1) (Supplementary Table S15), the best single combination was *minr* = 22, *minc* = 4 (Supplementary Table S17) but this produced clusters of only four to six phenotypes which lacked sensitivity 0.02 (M2)-0.24 (M1). No single combination of *minr* and *minc* parameters could detect all seven modules due to their diverse sizes (Supplementalry Table S16).

### 3.3 Performance of iBBiG on simulated meta-GSA dataset

**Fig. 3:**
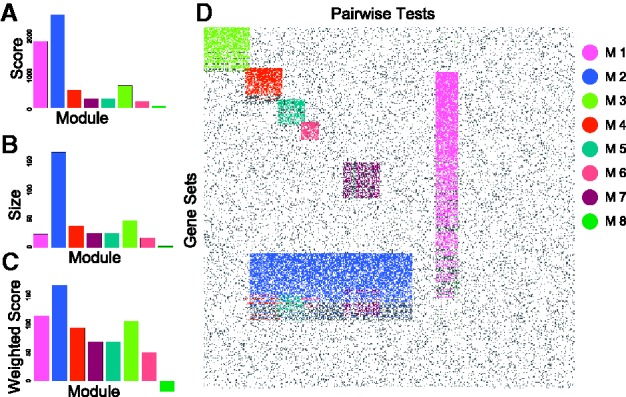
iBBiG bi-cluster analysis of the simulated dataset detects all modules (M1–M7) including those with overlaps (M3–M6). Plots show the **(A)** fitness score (*S*) **(B)** size (*k*) and **(C)** weighted score of the first eight modules. The weighted score is the *log*(*k*/m*S) where *m* is the total number of phenotypes. Modules M1–M7 are highlighted in **(D)** an image of the matrix in which rows and columns have the same order as Figure 1. The eighth module (M8) contains the residues of other modules which is reflected in a low weighted score

Next, we applied iBBiG to the simulated data (Fig. 3). iBBiG does not require the number of modules to be specified and instead extracts an excess number (for example, *nModules* = 20); the true number clusters are easily estimated from the cluster weighted score that reflects the size and fitness scores of each module. The modules are ranked in order of decreasing score (Fig. 3A). Only 7/20 modules had a fitness scores above background (Fig. 3A and C). The size of modules dramatically decreases after Module 7; only low scoring groups containing two or three phenotypes are found and these do not have positive weighted scorces. Because we do not remove the entire signal on each iteration, modules (which may be artifact) will arise from remaining signal residue from stronger modules. For example, phenotypes in M8 are a subset of those in M2. Overlaps in phenotypes and gene sets of M3-M6 are detected correctly.

To estimate the performance of iBBiG, it was applied to 100 randomly initialized artificial datasets (with *k* = 8) where it accurately assigned phenotypes to modules 98% of the time, with a specificity of 99%, precision of 96% and a sensitivity of 91%. These statistics are based on correct identification of phenotypes only and ignore gene set clustering (Supplementary Table S8). We found iBBiG detected gene sets membership with high specificity (99%) and precision (92%) but lacked some sensitivity (56%) following discretization of gene set scores (Supplementary Table S8). We do not expect to observe 100% sensitive detection of gene sets in modules M1, M2, M4 or M5 as these contain gene sets with less than 50% signal (Table 1). The best performing run of 100 detected almost all module phenotypes (specificity 100% and sensitivity 98%) and more gene sets (specificity 99.7% and sensitivity 72.5%) (Supplementary Table S9). The performance of iBBiG gene set prediction was also assessed by calculating of the variance of the scores within 100 runs (Supplementary Fig. 4) and calculating the ROC curves for each module on one run (Supplementary Fig. 5). The average runtime for the 400*x*400 matrix was 69.3 s on an Intel Core 2 Duo (3 GHz) running Windows XP.

### 3.4 iBBiG discovers 13 modules of breast cancer (GSVA analysis of three datasets)

**Fig. 4:**
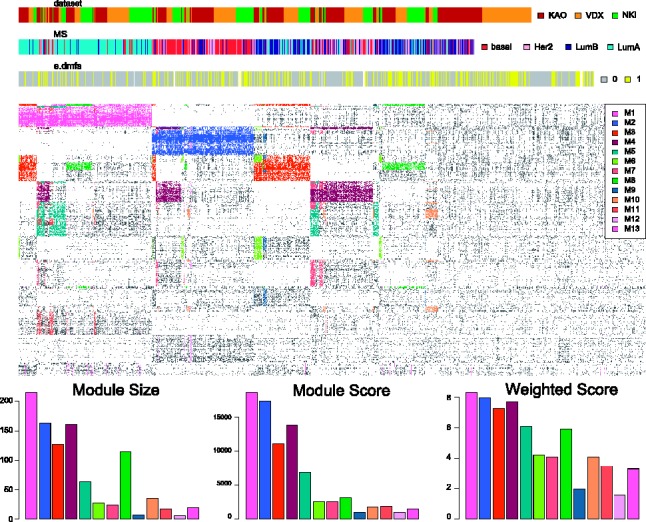
Meta-GSA using iBBiG bi-clustering of GSVA associations between 2459 gene sets which are up- or down-regulated (*n* = 5098) in gene expression profiles of 1008 primary breast cancer tissue samples, obtained using different array platforms in three independent studies (KAO, NKI and VDX). Phenotypes and the gene sets are ordered according to module membership in an image that highlights modules (*n* = 13) that had a weighted score greater than zero. Color bars above the plots indicate the study, molecular subtype and distant metastases free surival events of patients. These are brown (KAO)/yellow (VDX)/green (NKI)/red (basal-like)/pink (HER2 amplified)/Luminal B (navy)/Luminal A (cyan)/no distant metastases (gray) and distant metastases (yellow), respectively. The largest and first four modules were strongly associated with molecular subtype across studies being enriched in Luminal A (M1), Basal-like (M2), Luminal A or B (M3) and Basal-like or HER2 (M4) respectively. Further details are given in the text and in Supplementary Table S18. Plots below the image show the fitness size, score and weighted score of each modules

We can uncover gene sets associated with unknown clinical covariates using single sample GSA (e.g. GSVA). iBBiG is optimized to find overlapping bi-clusters in these complex data which can reveal new biological pathways associated with disease. When applied to 1008 gene expression profiles of primary breast tumors from three studies (Kao *et al.*, 2011; Minn *et al.*, 2007: van't Veer *et al.*, 2002) that had been obtained on different technological platforms each containing different numbers of features, iBBiG detected 13 modules with positive weighted scores (Figure 4). The first four modules (M1–M4) were strongly associated with breast cancer molecular subtypes Luminal A, Basal-like, Luminal A or B and Basal like or HER2, respectively (Fig. 4, Supplementary Table S18). Both M1 and M2 were enriched in cell proliferation genes and were highly predictive of tumor recurrence in all cancer subtypes and within Luminal A and Luminal B, respectively (Supplementary Table S18, Supplementary Figs. S11–18). Most other modules were subsets of samples in these modules but had different gene sets. For example, M9 contains different proliferative gene sets, and overlapped with M1 and M3. Although M11 overlapped with M4, it discovered a subset of poor prognosis basal-like patients and it was prognostic of metastases recurrence within the basal-like breast cancer (*P* < 0.01). M10 (Supplementary Fig. 19) was particularly interesting as it appears to predict patient prognosis within Luminal A and ERRB2 subtypes (DMFS, *P* < 0.001, Supplementary Figs. S20–22). There were 177 gene sets in M12 (Supplementary Fig. 19 and Table S19), which were associated with a stromal response to hypoxia and induction of angiogentic genes including TGFB1. The five top genes all contained transcription factor binding sites for V$ATF4_Q2 (GATHER, *P* < 0.0005) and 3/5 contained sites for V$OCT1_05 (GATHER, *P* < 0.0003) and are enriched in Gene Ontology terms GO:0045906 (negative regulation of vasoconstriction) and GO:0006701 (progesterone biosynthetic process), suggesting new avenues for research in these breast cancer subtypes.

### 3.5 iBBiG identifies eight modules associated with known clinical covariates in breast cancer

iBBiG was applied to extract 50 modules in a meta-GSA of known clinical covariates (GSEAlm) associated with 3875 gene expression profiles of breast tumors from 21 different studies obtained on diverse platforms that were available in GCOD (Liu *et al.*, 2011). It extracted eight modules (Fig. S4) which had between 9 and 43 pairwise tests (total n=448) (Supplementary Table 20). The largest, highest scoring module, B1, was among five modules (B1, B2, B4, B7 and B8) enriched in the phenotype (pairwise comparison) high versus low grade. Although all associated with tumor grade, gene sets in each module represented diverse biological actions; proliferation (B1 and B8), wound healing and cell–cell communication (B2), inflammatory processes and the tumor microenvironment (B4) and extracellular matrix (B7). Module 8 (B8) is a residue of the proliferation signal of B1 and all gene sets contained in B8 are also present in B1. Modules B3, B5 and B6 were associated with hormone receptor-positive luminal breast cancer. The Module B3 was associated with low grade cancer or normal tissue covariate pair tests and points towards the requirement of a high grade-cancer to dedifferentiate itself. The modules B5 and B6 were associated with protein processing (Supplementary Table S20 and Fig. S4).

We investigated two modules (B1 and B4) associated with p53 mutated, hormone receptor (ER-, PR-) negative, basal-like breast cancer—a poor prognosis subtype with few targeted therapies. While the importance of proliferation (B1) is well described, module B4 was of considerable biological interest (Supplementary Fig. 25) as it was characterized by immune and tumor microenvironment genes sets, including cytotoxic T-cell pathway, and the cytokine pathways IL-12 and IL-17. These genesets (B4) were associated with high-grade basal-like and luminal B tumors but were not associated with metastases (Supplementary Fig. S25). Survival analysis of a number of genes in this module in six publicly available datasets confirmed they were predictive of better outcome in Basal-like and ERBB2+ breast cancer (Supplementary Fig. S26).

## 4 DISCUSSION

The iBBiG bi-clustering algorithm is optimized for module discovery in sparse noisy binary genomics data and can be used for meta-GSA of multiple genomics datasets, to discover modules: groups of phenotypes whose differential gene expression profiles are enriched in the same gene sets. Data integration is made tractable by transformation of continuous ‘noisy’ gene expression data (with different probes/genes in each study) into profiles of differentially enriched gene sets (which are common to all studies). We examined two GSA approaches, GSEAlm which tests for enrichment of gene sets in genes that are differentially regulated between conditions and GSVA which is single sample GSA. The former can be applied to identifying gene sets or pathways associated with known clinical covariates, the latter is a pure discovery approach that ignores prior sample knowledge.

We designed iBBiG to have high specificity and thereby minimize the false-positive rate when discovering new classes, but the iterative approach employed in iBBiG ensures it is sufficiently sensitive to discover weak signals, even if they are potentially masked by stronger ones. When applied to simulated data it outperforms widely used global clustering approaches (*K*-means, hierarchical cluster analysis) and newer bi-clustering approaches (Bimax, FABIA and COALESCE) and is able to find overlapping gene set modules of varying sizes. iBBiG was able to identify all clusters in a simulated meta-GSA dataset with high levels of specificity and sensitivity. An advantage of iBBiG relative to other methods is that it does not require *a priori* knowledge of the true number of clusters. Following the application of iBBiG, the number of true clusters can be estimated from the weighed scores of the extracted modules. In some cases, we observed that a module may represent the residue or remaining signal of a stronger, previously extracted module. This residue remains because iBBiG only removes information from the data matrix that is actually used for the entropy-based score in a module. However, we do not consider these residual modules to be a shortcoming of the method as their existence facilitates discovery of the true overlap between modules and, further, these modules can be easily detected by looking at the overlap of phenotypes and gene sets.

Although iBBiG includes several parameters, we have shown that most impact only computation time and do not effect cluster discovery. The only parameter that had an impact on cluster discovery was α, which regulates the weighting between cluster homogeneity and the number of phenotypes. This parameter is useful in fine-adjustment of the sensitivity–specificity ratio.

One major advantage of iBBiG is its robustness in the presence of noise and its tolerance of missing data. It can tolerate high levels of noise as the entropy derived fitness score add members to a bi-cluster once the number of associations exceeds 50%. We demonstrate that iBBiG performs well even in the presence of false-positive associations and noise in both signal (20%) and background (40%). Second, iBBiG does not require a gene set to be associated with all phenotypes in a bi-cluster which is a attractive feature in complex biological data were biological processes maybe redundant or regulated by multiple factors concurrently. Many other bi-clustering algorithms, including Bimax and the recently described BiBiT (Rodriguez-Baena *et al.*, 2011), discover only homogenous bi-clusters and have low tolerance to noise and missing data. Bimax identified a large number of mini-bi-clusters and was unable to identify large clusters in our simulated dataset. In practice, application of Bimax to genomics data requires post-processing of bi-cluster results in order to either join or visualize overlapping bi-clusters (Santamaria *et al.*, 2008).

We applied iBBiG to discovery of new modules among 1008 primary breast tumors and discovered 13 modules in an iBBiG-GSVA analysis. Each module contained samples from multiple studies demonstrating successful data integration. While the largest highest confidence modules (M1–M4) discovered breast cancer molecular subtypes known to be important in breast cancer, the smaller modules represented sub-sets of these subtypes, supporting recent evidence that there are subtypes within each of the principal breast cancer molecular subtypes (Curtis *et al.*, 2012). The module M10 was characterized by gene sets associated with angiogenesis in response to hypoxia (or HIF1A degregulation) and was a strong predictor of recurrence in Luminal and ERRB2 amplied breast cancer. We uncovered different modules (*n* = 8) associated with pairwise tests of breast cancer clinical covariates in a meta-GSA of 21 breast cancer gene expression datasets. Five of the eight modules including the first and largest module was strongly associated with tumor grade. Most high grade tumors were characterized by increased cell cycle (B1/B8) and those with fewer metastases had significant regulation of immune response genes (B4). In a meta-analysis of two datasets, Shi *et al.*, (2010) also reported an up-regulation of proliferation genes and down-regulation of cell adhesion genes in high-grade breast tumors. Although they had insufficient numbers of patients to establish statistical significance, they observed that high levels of immune genes were an indicator of good prognosis in high-grade breast cancer patients. Our analysis also suggests B4 immune response is associated with better outcome. When we examined which genes that most frequently appeared in module B4 GeneSigDB gene signatures, we found several chemokines including CCL5 (RANTES), a key regulators of T-cell immune response highly expressed in breast cancer and reported to be associated with metastases and progression (Soria and Ben-Baruch, 2008; Zhang *et al.*, 2009). However, our analysis does not fit this prevailing hypothesis and suggests CCL5 is associated with good prognosis in high-grade breast cancer patients.

In this study, we have used iBBiG to discover clusters in matrices of discretized *P*-values from GSA of gene expression data; however, the method can also be easily applied to GSA of other different data types including SNP data (Cantor *et al.*, 2010; Raychaudhuri *et al.*, 2009). iBBiG can be applied to non-geneset data. For example, to demonstrate the application of iBBiG to an extremely sparse matrix (<0.3% > 0) in which small clusters are expected, iBBiG was applied to discretized data from the NHGRI genome-wide association study (GWAS) catalog (Hindorff *et al.*, 2009). As the weighted scores were low for modules identified, we averaged results over 100 runs of iBBiG and chose 10 robust modules. Only genes and traits that occured in at least 65/100 runs were included (Supplementary Fig. S27). These modules are provided in Supplementary Table S22. It discovered a possible link between triglyercides, HDL cholesterol and waist circumference with genes GCKR, LPL, BUD13 and ZNF259. Although LPL, BUD13 and ZNF259 has been implicated previously, this module suggested a new link with an expanding waistline and GCKR. While GSA requires input gene sets, it is not restricted to databases of curated gene sets and can use gene sets deduced through text mining from the published literature (Jelier *et al.*, 2011; Krallinger *et al.*, 2010; Raychaudhuri *et al.*, 2009). We anticipate iBBiG will be useful in integrated data analysis of multiple data types. iBBiG can be performed on any binary matrix and could be applied to binary protein–protein interaction or RNAi data; we would like to extend it to other data types, including categorical data. An attractive feature of iBBiG compared to others methods such as the recently described logistic regression meta-GSA approach (Montaner and Dopazo, 2010) is its ability to perform integrative analysis using dozens of datasets.

In summary, iBBiG provides a simple, robust, rapid and scalable method for meta-GSA. When applied to simulated data it outperforms commonly used clustering and bi-clustering approaches and iteratively discovers gene set modules made up of both strong and weaker signals. Meta-GSA using iBBiG constitutes a new approach for discovery of pathway and gene set behavior across multiple studies and provides a higher-level understanding of gene and cellular function.

*Funding*: This work was supported the Claudia Adams Barr foundation and grant 1U19CA148065 from the National Cancer Institute of the US National Institutes of Health. We are grateful to Prof. Curtis Huttenhower for his invaluable expertise and generous help in running COALESCE. We thank Benjamin Haibe-Kains and Stephan Winkler for valuable comments during the development of the algorithm, Markus Schröder for offering his expertise on implementing the R functions as C libraries and Prof. Daniel Silver for assistance in biological interpretation of results of the analyses.

*Conflict of Interest*: none declared

## Supplementary Material

Supplementary Data

Supplementary Data
