# Supplementary material for: iBBiG: iterative binary bi-clustering of gene sets
Source: Bioinformatics. 2012 Jul 12;28(19):2484–92. doi: 10.1093/bioinformatics/bts438 (PMC3463116; doi:10.1093/bioinformatics/bts438)
Supplement: Supplementary Data [file supp_28_19_2484__index.html]

iBBiG: iterative binary bi-clustering of gene sets — Supplementary Data 

# iBBiG: iterative binary bi-clustering of gene sets

## Supplementary Data

files

**Files in this Data Supplement:**

- Supplementary Data - pdf file
