# Supplementary material for: iBBiG: iterative binary bi-clustering of gene sets
Source: Bioinformatics. 2012 Jul 12;28(19):2484–92. doi: 10.1093/bioinformatics/bts438 (PMC3463116; doi:10.1093/bioinformatics/bts438)
Supplement: Supplementary Data [file supp_bts438_iBBiG_supplement.pdf]

1 SUPPLEMENT

iBBiG: Iterative Binary Bi-clustering of Gene Sets

Daniel Gusenleitner, Eleanor A Howe, Stefan Bentink, John Quackenbush and Aedín C Culhane

Contact Information:
Aedin Culhane: aedin@jimmy.harvard.edu
Daniel Gusenleitner: gusef@bu.edu

2 OPTIMIZATION AND EVALUATION:

Details of the 21 breast cancer datasets analyzed using GSEAlm and iBBiG bi-cluster discovery

Table 1. 21 Breast cancer datasets that are included in the study

Identifier	Array Type	#CELs	Pubmed	First Author	Journal	PubDate	Title
breast_boersma	HG-U133A	95	17999412	BJ Boersma	Int J Cancer	Mar 08	A stromal gene signature associated with inflammatory breast cancer.
breast_chang	HG-U95Av2	24	12907009	JC Chang	Lancet	Aug 03	Gene expression profiling for the prediction of therapeutic response to docetaxel in patients with breast cancer.
breast_chen	HG-U133 Plus 2	185	19266279	DT Chen	Breast Cancer Res Treat	Mar 09	Proliferative genes dominate malignancy-risk gene signature in histologically-normal breast tissue.
breast_chin	U133AAofAv2	130	17157792	K Chin	Cancer Cell	Dec 06	Genomic and transcriptional aberrations linked to breast cancer pathophysiologies.
breast_farmer	HG-U133A	49	15897907	P Farmer	Oncogene	Jul 05	Identification of molecular apocrine breast tumours by microarray analysis.
breast_fournier	HG-U133A	12	16849555	MV Fournier	Cancer Res	Jul 06	Gene expression signature in organized and growth-arrested mammary acini predicts good outcome in breast cancer.
breast_huang	HG-U95Av2	89	12747878	E Huang	Lancet	May 03	Gene expression predictors of breast cancer outcomes.
breast_ivshina	HG-U133A, HG-U133B	578	17079448	AV Ivshina	Cancer Res	Nov 06	Genetic reclassification of histologic grade delineates new clinical subtypes of breast cancer.
breast_loi	HG-U133A, HG-U133B, HG-U133 Plus 2	741	17401012	S Loi	J Clin Oncol	Apr 07	Definition of clinically distinct molecular subtypes in estrogen receptor-positive breast carcinomas through genomic grade.
breast_loi1	HG-U133 Plus 2	77	18498629	S Loi	BMC Genomics	2008	Predicting prognosis using molecular profiling in estrogen receptor-positive breast cancer treated with tamoxifen.
breast_lu	HG-U133 Plus 2	127	18297396	X Lu	Breast Cancer Res Treat	Mar 08	Predicting features of breast cancer with gene expression patterns.
breast_miller	HG-U133A, HG-U133B	502	16141321	LD Miller	Proc Natl Acad Sci	Sep 05	An expression signature for p53 status in human breast cancer predicts mutation status, transcriptional effects, and patient survival.
breast_minn	HG-U133A	121	16049480	AJ Minn	Nature	Jul 05	Genes that mediate breast cancer metastasis to lung.
breast_pawitan	HG-U133A, HG-U133B	318	16280042	Y Pawitan	Breast Cancer Res	2005	Gene expression profiling spares early breast cancer patients from adjuvant therapy: derived and validated in two population-based cohorts.
breast_richardson	HG-U133 Plus 2	47	16473279	AL Richardson	Cancer Cell	Feb 06	X chromosomal abnormalities in basal-like human breast cancer.
breast_schmidt	HG-U133A	200	18593943	M Schmidt	Cancer Res	Jul 08	The humoral immune system has a key prognostic impact in node-negative breast cancer.
breast_seitz	HG-U133A	26	17410534	A Klein	Int J Cancer	Aug 07	Comparison of gene expression data from human and mouse breast cancers: identification of a conserved breast tumor gene set.
breast_sotiriou	HG-U133A	189	16478745	C Sotiriou	J Natl Cancer Inst	Feb 06	Gene expression profiling in breast cancer: understanding the molecular basis of histologic grade to improve prognosis.
breast_turashvili	HG-U133 Plus 2	30	17389037	G Turashvili	BMC Cancer	2007	Novel markers for differentiation of lobular and ductal invasive breast carcinomas by laser microdissection and microarray analysis.
breast_wang	HG-U133A	286	15721472	Y Wang	Lancet		Gene-expression profiles to predict distant metastasis of lymph-node-negative primary breast cancer.
breast_west	Hu6800	49	11562467	M West	Proc Natl Acad Sci	Sep 01	Predicting the clinical status of human breast cancer by using gene expression profiles.

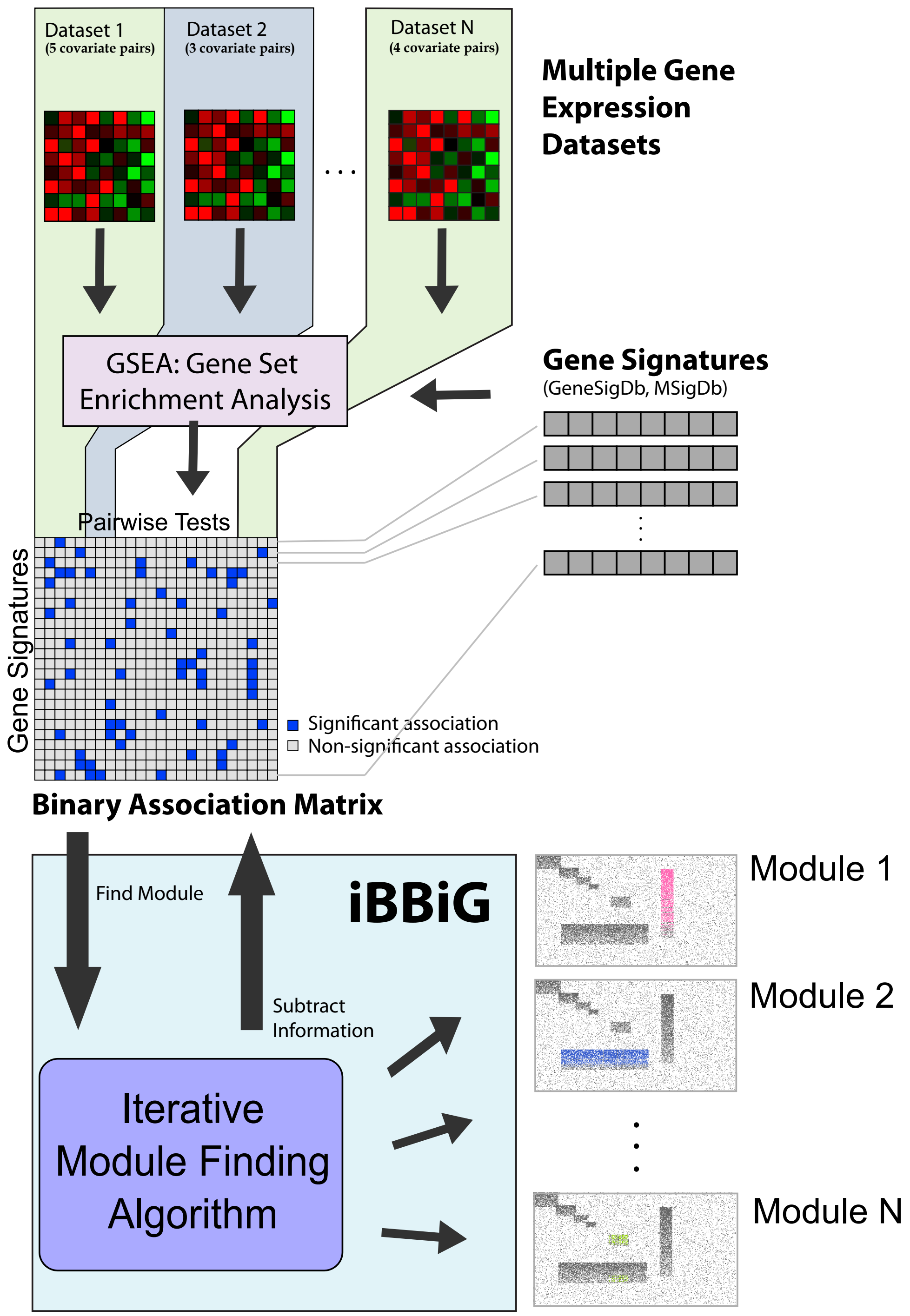

Supplementary Fig. 1. Flowchart of iBBiG meta-Gene Set analysis. Results of single sample or pairwise test gene set enrichment analysis (GSA) are descretized and subject to iBBiG cluster discovery which iteratively find and masks clusters in the binary data matrix

3 OPTIMIZATION AND EVALUATION:

Different values of each parameter were tested to determine an optimal value. Values of α-parameter from [0.1, 0.9] in 0.1 increments were tested, each using 100 randomly generated artificial datasets. Similar tests were performed for the selection pressure [1.1, 1.9] in 0.1 increments, population sizes of 50, 100, 150, 250, and 300, success ratios [0.1, 0.9] in 0.1 increments and mutation rates [0.02, 0.20] in 0.02 increments.

The behavior of the GA iBBiG was also tested with differing levels of background noise, where the noise was increased in 5% increments, up to 50%. To evaluate the performance of each of the algorithms tested, including the various parameter values considered, the extracted modules were compared to the actual clusters in the artificial dataset. The results were used to build a contingency table and to calculate the sensitivity and specificity.

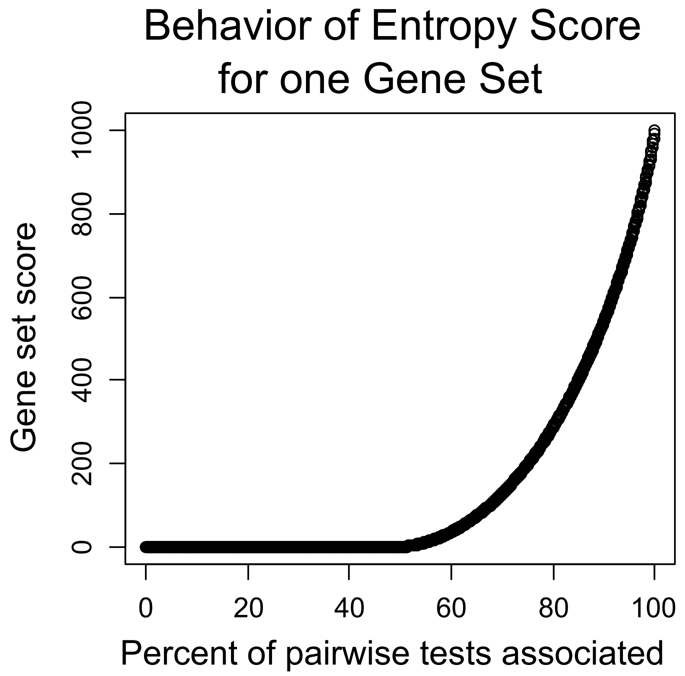

Supplementary Fig. 2. Behavior of the entropy based score for one single gene set and 1000 clinical phenotypes. The left side represents a situation in which the gene set is not associated with any of the phenotypes within the chosen grouping, whereas the right side indicates a strong association with all phenotypes.

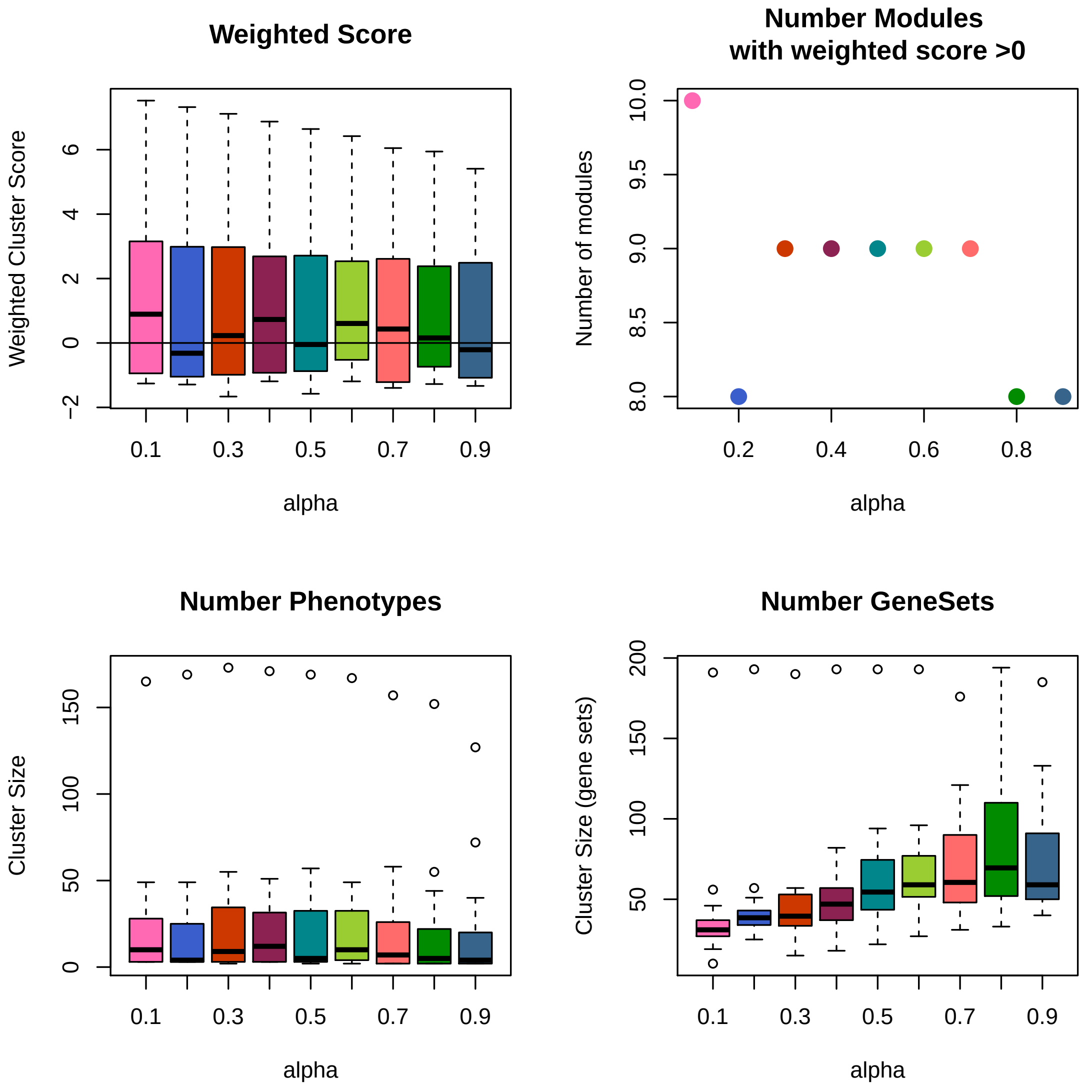

Supplementary Fig. 3. Effect of changes to alpha (0.1-0.9) on A) weighted score, B) number of modules with a weighted score ¿0 C) mean number of phenotypes and D) mean number of gene sets per modules when applied to simulated data. All iBBiG parameters were run at default with the exception of alpha. The nModules was 20.

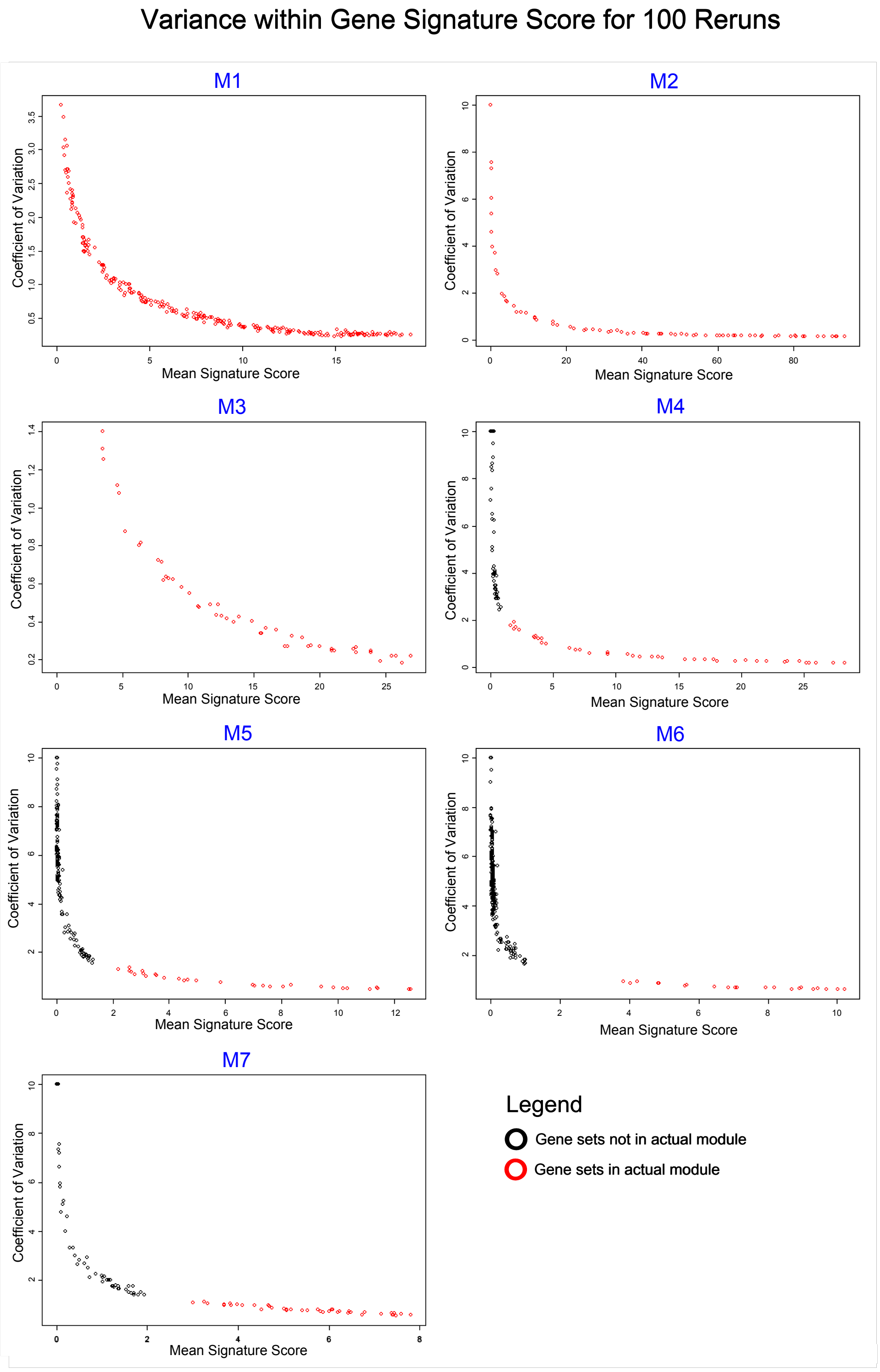

Supplementary Fig. 4. True gene sets scored consistently high scores and had a low coefficient of variation in 100 reruns of iBBiG applied to the simulated dataset. Plot shows variance of gene set scores in M1-M7 in the artificial dataset. Note the Y and X axis scales vary in plots M1-M7. Gene signatures with low scores have higher co-efficient of variation indicating that they occurred inconsistently in 100 rerun of iBBiG. This plot also demonstrates the usefulness of scoring of gene sets, as gene sets with higher scores are likely to be more robustly associated with a module

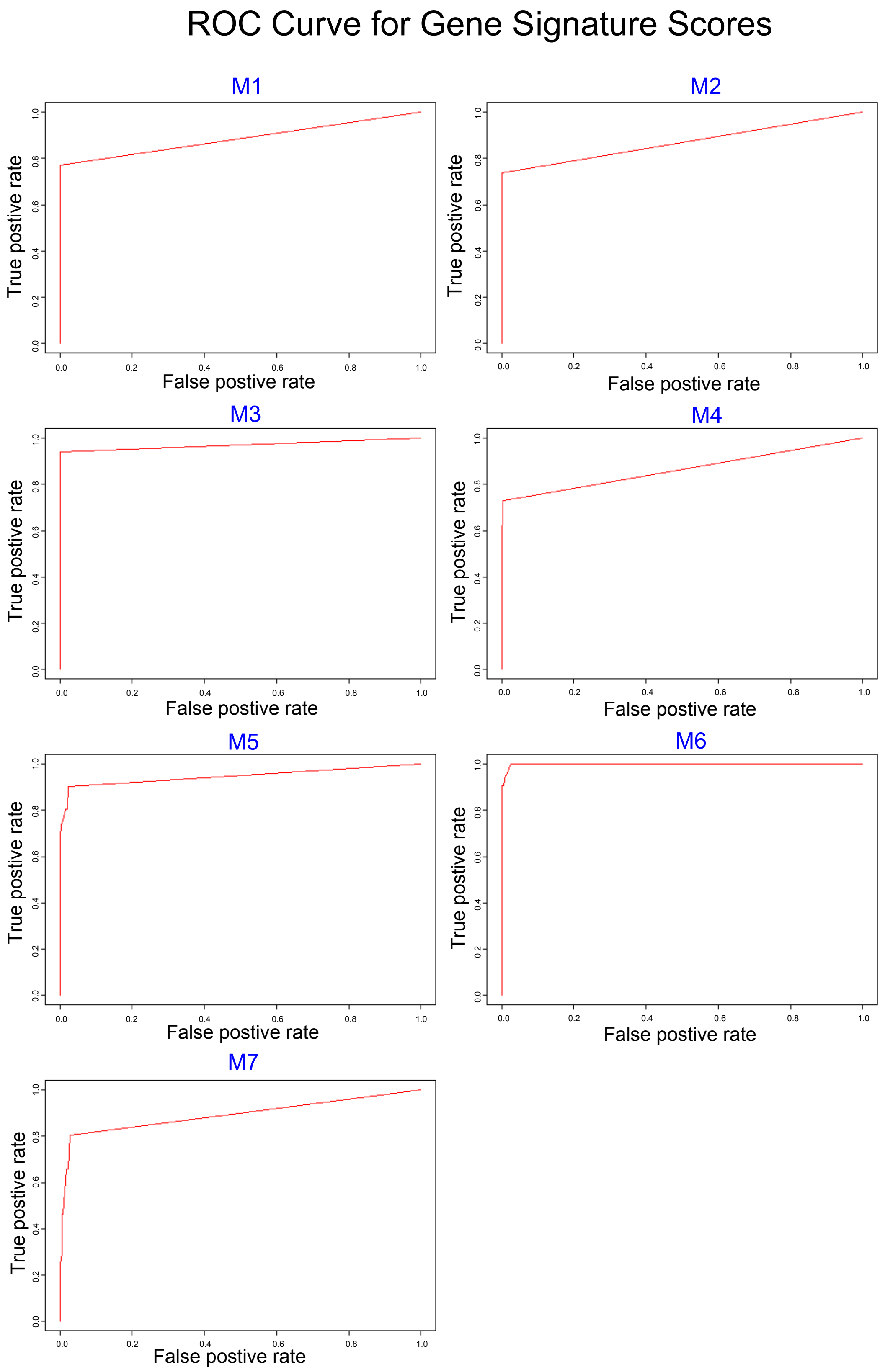

Supplementary Fig. 5. ROC curve of gene set score prediction in M1-M7 in the artificial dataset resulting from one run using iBBiG.

Table 2. Test of different weightings between homogeneity and number of phenotypes for the entropy score (alpha-parameter). The population size for all tests was set to 100, the mutation rate to 0.08, the success ratio for the offspring selection to 0.6 and the selection pressure for the parent selection to 1.2. The alpha parameter was varied between 0.1 (larger size, lower homogeneity) and 0.9 (smaller size,higher homogeneity) . In order to get more stable results 100 artificial datasets were created and the results were combined.

Alpha	0.1	0.2	0.3	0.4	0.5	0.6	0.7	0.8	0.9
Accuracy	97.3%	98.6 %	98.7%	98.5%	980%	96.8%	98.3%	95.2%	94.5%
Precision	94.8%	97.5%	98.0%	98.0%	97.5%	97.1%	97.2%	97.2%	97.2%
Specificity	99.2%	99.6%	99.7%	99.7%	99.6%	99.6%	99.7%	99.7%	99.7%
Sensitivity	85.0%	92.5%	92.6%	90.5%	87.4%	78.9%	97.8%	66.9%	61.1%

Table 3. Test of different population sizes. The mutation rate was set to 0.08, the success ratio for the offspring selection to 0.6, the selection pressure for the parent selection to 1.2 and the alpha parameter for the weighting of the homogeneity was set to 0.3. The population size was varied between 50 and 100. To improve stability, 100 artificial datasets were created and the results were combined.

Population	15	25	35	50	100	150
Accuracy	97.0%	97.8%	98.1%	98.6%	98.8%	98.9%
Precision	95.3%	96.7%	97.3%	97.6%	98.0%	98.1%
Specificity	99.4%	99.5%	99.6%	99.6%	99.7%	99.7%
Sensitivity	81.6%	86.5%	88.3%	91.7%	93.2%	93.8%

Table 4. Test of different mutation rates for the recombination operator of the genetic algorithm. The population size for all tests was set to 100, the success ratio for the offspring selection to 0.6, the selection pressure for the parent selection to 1.2 and the alpha parameter for the weighting of the homogeneity was set to 0.3. The mutation rate was varied between 0.02 and 0.2. To improve stability, 100 artificial datasets were created and the results were combined.

Mutation	0.02	0.04	0.06	0.08	0.10	0.12	0.14	0.16	0.18	0.20
Accuracy	98.4%	98.8%	98.8%	98.9%	98.7%	99.0%	98.7%	98.8%	98.7%	98.7%
Precision	97.7%	98.0%	98.0%	98.1%	98.0%	98.1%	97.9%	98.0%	97.9%	97.8%
Specificity	99.7%	99.7%	99.7%	99.7%	99.7%	99.7%	99.7%	99.7%	99.7%	99.7%
Sensitivity	90.2%	93.1%	92.9%	92.5%	92.4%	94.3%	92.5%	93.3%	92.5%	99.2%

Table 5. Test of different values for the selection pressure of the parent selection operator of the genetic algorithm. The population size for all tests was set to 100, the mutation rate was set to 0.08, the success ratio for the offspring selection to 0.6 and the alpha parameter for the weighting of the homogeneity was set to 0.3. The selection pressure was varied between 1.2 and 1.8. To improve stability, 100 artificial datasets were created and the results were combined.

SP	1.1	1.2	1.3	1.4	1.5	1.6	1.7	1.8	1.9
Accuracy	98.2%	98.8%	98.8%	98.9%	99.0%	98.9%	98.8%	99.0%	98.7%
Precision	97.9%	98.1%	98.0%	98.1%	98.2%	98.0%	97.9%	98.2%	98.1%
Specificity	99.7%	99.7%	99.7%	99.7%	99.7%	99.7%	99.7%	99.7%	99.7%
Sensitivity	93.1%	93.1%	93.3%	93.7%	94.1%	94.0%	93.4%	94.2%	93.9%

Table 6. Test of different values for success ratio of the offspring selection operator of the genetic algorithm. The population size for all tests was set to 100, the mutation rate was set to 0.08, the selection pressure for the parent selection to 1.2 and the alpha parameter for the weighting of the homogeneity was set to 0.3. The success ratio was varied between 0.2 and 0.6. To improve stability, 100 artificial datasets were created and the results were combined.

SR	0.1	0.2	0.3	0.4	0.5	0.6	0.7	0.8	0.9
Accuracy	96.7%	96.8%	97.0%	97.7%	98.5%	98.8%	98.8%	99.0%	98.9%
Precision	97.3%	97.3%	97.5%	97.6%	97.6%	97.9%	97.9%	98.2%	98.0%
Specificity	99.7%	99.7%	99.7%	99.7%	99.6%	99.7%	99.7%	99.7%	99.7%
Sensitivity	77.8%	78.4%	80.0%	85.3%	97.0%	93.0%	93.4%	94.4%	94.0%

Table 7. Sensitivity and specificity of all modules contained in the artificial dataset using varying levels of noise. The enumeration of the modules corresponds to the enumeration in Figure 1. The signal strength of each single module is stated in the first line. The higher number indicates the signal strength on the top, the lower, the signal strength on the bottom of a module. Background noise levels between 0 and 50% were tested. For each noise level the clustering was repeated 100 times, the modules in the single iterations were matched to the actual modules and the specificity and sensitivity were calculated for all of them. Results, where either sensitivity or specificity dropped under 50% are in grey. An alpha of 0.5, a selection pressure of 1.2, a population size of 100, a mutation rate of 0.8 and a success ratio of 0.6 was used for the GA.

	Mod 1		Mod 2		Mod 3		Mod 4		Mod 5		Mod 6		Mod 7	
Dimension	25	250	175	75	50	50	40	40	30	30	20	20	40	40
Signal	0.4	0.9	0.4	0.8	0.5	0.8	0.4	0.9	0.4	0.8	0.6	0.9	0.5	0.6
Noise	Spec	Sens	Spec	Sens	Spec	Sens	Spec	Sens	Spec	Sens	Spec	Sens	Spec	Sens
0.00	100	99.5	100	99.6	97.7	69.8	100	94.0	98.8	84.2	99.8	79.3	99.8	66.0
0.05	100	100	99.7	97.9	100	91.2	100	96.3	98.9	81.9	100	89.7	99.9	67.1
0.10	100	100	99.7	97.7	100	96.7	100	97.0	98.8	83.2	100	95.5	99.8	65.2
0.15	100	100	99.7	97.4	100	97.2	100	96.7	98.8	81.3	100	87.5	99.8	66.0
0.20	100	100	99.7	97.7	100	97.0	100	96.1	98.7	79.5	99.9	85.5	99.6	62.8
0.25	100	100	99.7	97.8	100	96.6	100	96.4	99.1	45.3	99.7	62.7	99.6	40.3
0.30	100	100	99.6	97.8	100	96.4	99.8	95.6	99.1	22.7	99.2	49.1	99.5	25.7
0.35	100	100	99.4	98.7	99.3	95.2	99.1	93.2	98.9	11.6	98.6	30.0	98.9	14.7
0.40	99.4	100	93.2	92.2	94.8	87.1	94.0	46.6	95.6	11.8	95.6	16.1	95.3	12.1
0.45	94.7	100	81.1	83.7	86.7	46.8	89.7	17.7	90.0	10.5	90.0	12.0	89.7	13.8
0.50	84.1	57.7	51.1	80.7	84.3	29.4	84.2	23.5	84.5	21.6	84.5	22.5	84.0	22.5

Table 8. Average results of iBBiG analyses on artificial dataset. Default parameters; alpha of 0.3, a selection pressure of 1.2, a population size of 100, a mutation rate of 0.8 and a success ratio of 0.6 was used for the GA. 100 runs of iBBiG were performed to test the robustness of iBBiG. Results of the best run are given in Table 9. Accur, Sens and Spec are accuracy, sensitivity and specificity respectively.

Module	JI	Cluster Size		phenotype (Col)					Gene Set (Row)				
		nRow	nCol	Accur	Sens	Spec	PPV	NPV	Accur	Sens	Spec	PPV	NPV
M1	0.99	114.88	24.73	1.00	0.99	1.00	1.00	1.00	0.66	0.46	1.00	1.00	0.53
M2	0.98	39.04	173.79	0.99	0.99	1.00	1.00	0.99	0.91	0.52	1.00	1.00	0.90
M3	0.99	33.40	49.34	1.00	0.99	1.00	1.00	1.00	0.96	0.67	1.00	1.00	0.95
M4	0.97	22.57	39.41	1.00	0.97	1.00	0.99	1.00	0.95	0.56	1.00	0.99	0.95
M5	0.82	19.80	34.96	0.97	0.87	0.98	0.95	0.99	0.96	0.54	0.99	0.87	0.96
M6	0.70	19.42	36.96	0.94	0.82	0.95	0.76	0.99	0.96	0.57	0.98	0.70	0.98
M7	0.74	27.19	29.82	0.97	0.74	1.00	1.00	0.97	0.95	0.58	0.99	0.86	0.96

Table 9. The best performance of iBBiG in results of Table 8 100 runs. The run with the highest JI to phenotype (columns) is reported. (The same approach to select the best results was used in Table reftable:BimaxBest. Accur. Sens amd Spec are accuracy, sensitivity and specificity respectively

Module	JI	Cluster Size		phenotype (Col)					Gene Set (Row)				
		nRow	nCol	Accur	Sens	Spec	PPV	NPV	Accur	Sens	Spec	PPV	NPV
M1	1.00	137	25	1.00	1.00	1.00	1.00	1.00	0.72	0.55	1.00	1.00	0.57
M2	0.99	46	173	0.99	0.99	1.00	1.00	0.99	0.93	0.61	1.00	1.00	0.92
M3	1.00	42	50	1.00	1.00	1.00	1.00	1.00	0.98	0.84	1.00	1.00	0.98
M4	1.00	26	40	1.00	1.00	1.00	1.00	1.00	0.96	0.65	1.00	1.00	0.96
M5	0.97	23	29	1.00	0.97	1.00	1.00	1.00	0.97	0.70	0.99	0.91	0.98
M6	1.00	19	20	1.00	1.00	1.00	1.00	1.00	1.00	0.95	1.00	1.00	1.00
M7	0.90	35	36	0.99	0.90	1.00	1.00	0.99	0.97	0.78	0.99	0.89	0.97

4 COMPARISON TO OTHER METHODS:

To visualize the bi-clustering results of iBBiG, FABIA, COALESCE, Hierarchical clustering and k-means clustering, the artificial dataset was superimposed with the extracted clustering of phenotypes and gene signatures, without changing the order of rows or columns. The exception was hierarchical clustering which is a global method that does not determine specific clusters; hence the resulting order of gene sets and phenotypes was used and the actual modules color-coded as they were in the artificial dataset. The rows and columns of the breast cancer dataset were rearranged so that the largest module is in the upper left corner, followed diagonally by the second largest and so forth. Overlapping phenotypes or gene sets are shown in the first module they occur in, but in the color coding of the last module they are found to show the overlaps between modules.

Table 10. Result of Hierarchical Clustering using binary distance and Ward's minimum variance clustering in both directions. The dendrogram was cut to give 6 clusters, as K=6 was found to be the minimal number to give maximum average JI to clusters M1-M7. Accur. Sens amd Spec are accuracy, sensitivity and specificity respectively.

Module	Best Cluster	JI	Cluster Size		phenotype (Col)					Gene Set (Row)				
			nRow	nCol	Accur	Sens	Spec	PPV	NPV	Accur	Sens	Spec	PPV	NPV
M1	6	1.00	75.00	25.00	1.00	1.00	1.00	1.00	1.00	0.44	0.20	0.83	0.67	0.38
M2	3	0.57	30.00	100.00	0.81	0.57	1.00	1.00	0.75	0.74	0.00	0.91	0.00	0.80
M3	1	1.00	48.00	50.00	1.00	1.00	1.00	1.00	1.00	0.99	0.96	1.00	1.00	0.99
M4	2	0.85	79.00	36.00	0.98	0.88	1.00	0.97	0.99	0.70	0.00	0.78	0.00	0.88
M5	3	0.24	30.00	100.00	0.80	0.83	0.80	0.25	0.98	0.85	0.00	0.92	0.00	0.92
M6	3	0.20	30.00	100.00	0.80	1.00	0.79	0.20	1.00	0.88	0.00	0.92	0.00	0.95
M7	4	0.97	128.00	39.00	1.00	0.97	1.00	1.00	1.00	0.58	0.00	0.64	0.00	0.85

Table 11. Results of COALESCE with default parameters. JI between columns and rows are provided. Accur. Sens amd Spec are accuracy, sensitivity and specificity respectively. 5 clusters were identified and the cluster which had the highest JI to each module is listed.

Module	Best Cluster	JI	Cluster Size		phenotype (Col)					Gene Set (Row)				
			nRow	nCol	Accur	Sens	Spec	PPV	NPV	Accur	Sens	Spec	PPV	NPV
M1	1	0.32	50	74	0.87	0.96	0.87	0.32	1.00	0.25	0.00	0.67	0.00	0.29
M2	4	0.99	75	176	1.00	1.00	1.00	0.99	1.00	1.00	1.00	1.00	1.00	1.00
M3	1	0.68	50	74	0.94	1.00	0.93	0.68	1.00	1.00	1.00	1.00	1.00	1.00
M4	2	0.67	38	60	0.95	1.00	0.94	0.67	1.00	0.99	0.95	1.00	1.00	0.99
M5	3	0.70	36	43	0.97	1.00	0.96	0.70	1.00	0.98	0.97	0.98	0.81	1.00
M6	4	0.11	75	176	0.61	1.00	0.59	0.11	1.00	0.76	0.00	0.80	0.00	0.94
M7	5	0.95	60	42	0.99	1.00	0.99	0.95	1.00	0.95	1.00	0.94	0.67	1.00

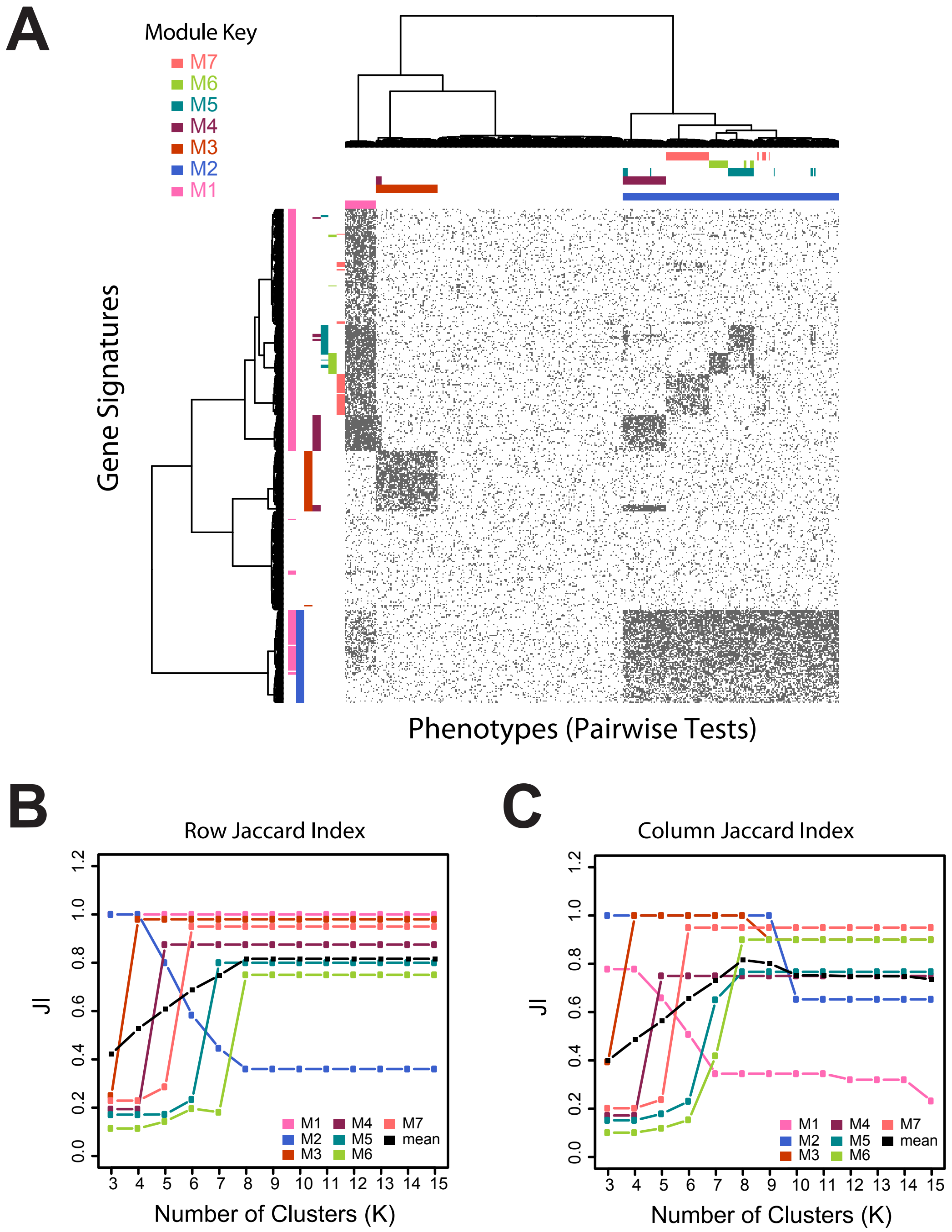

Supplementary Fig. 6. Hierarchical clustering of the simulated dataset showing A) heatmap and dengrogram of results in which the color bars highlight the true modules. The dendrogram was cut to give 3-15 cluster (K=3:15) and the Jaccard Index of each module to the true modules was calculated for each K. Plots show the maximum Jaccard Index of the best cluster to each B) row (gene set) or C) column (phenotype) of true clusters for each K

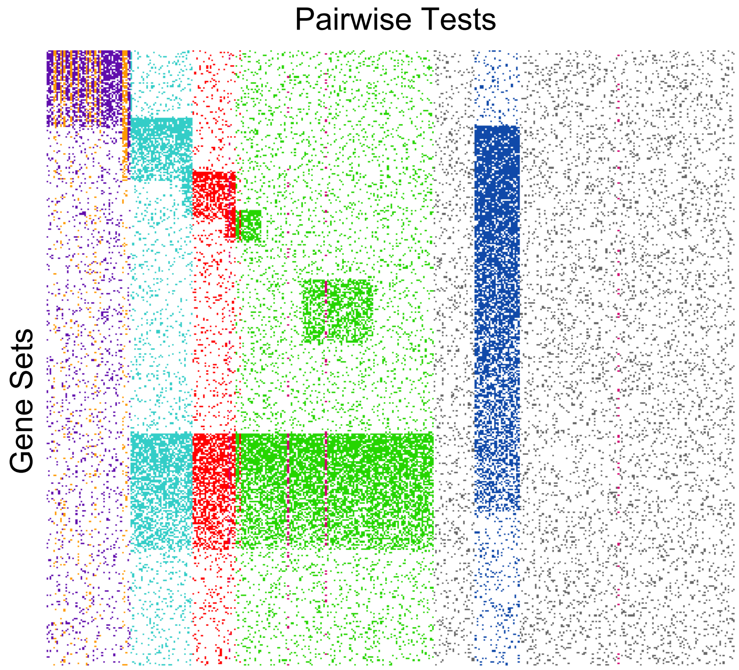

Supplementary Fig. 7. K-means clustering of the artificial dataset using 7 centers and applied only to phenotypes. Since this method clusters only in one dimension it is not able to handle overlaps of phenotypes.

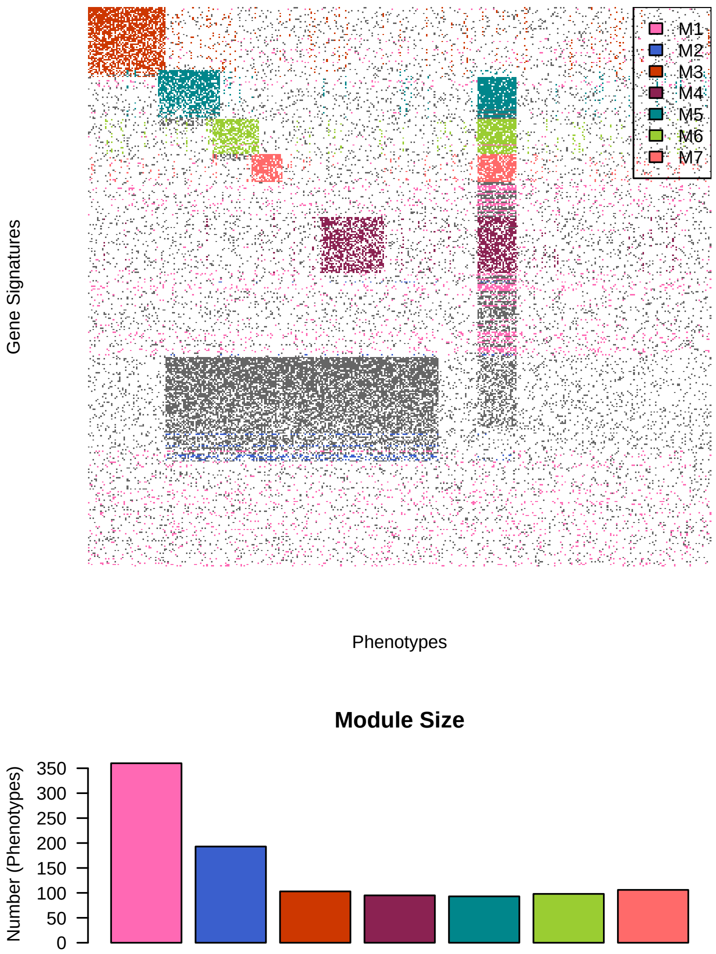

Supplementary Fig. 8. FABIA bi-clustering determined most of the smaller modules (M3, M5-M7) correctly, but also produced large modules which lacked specificity; M1 contained 360/400 phenotypes in it and high number of false positives.

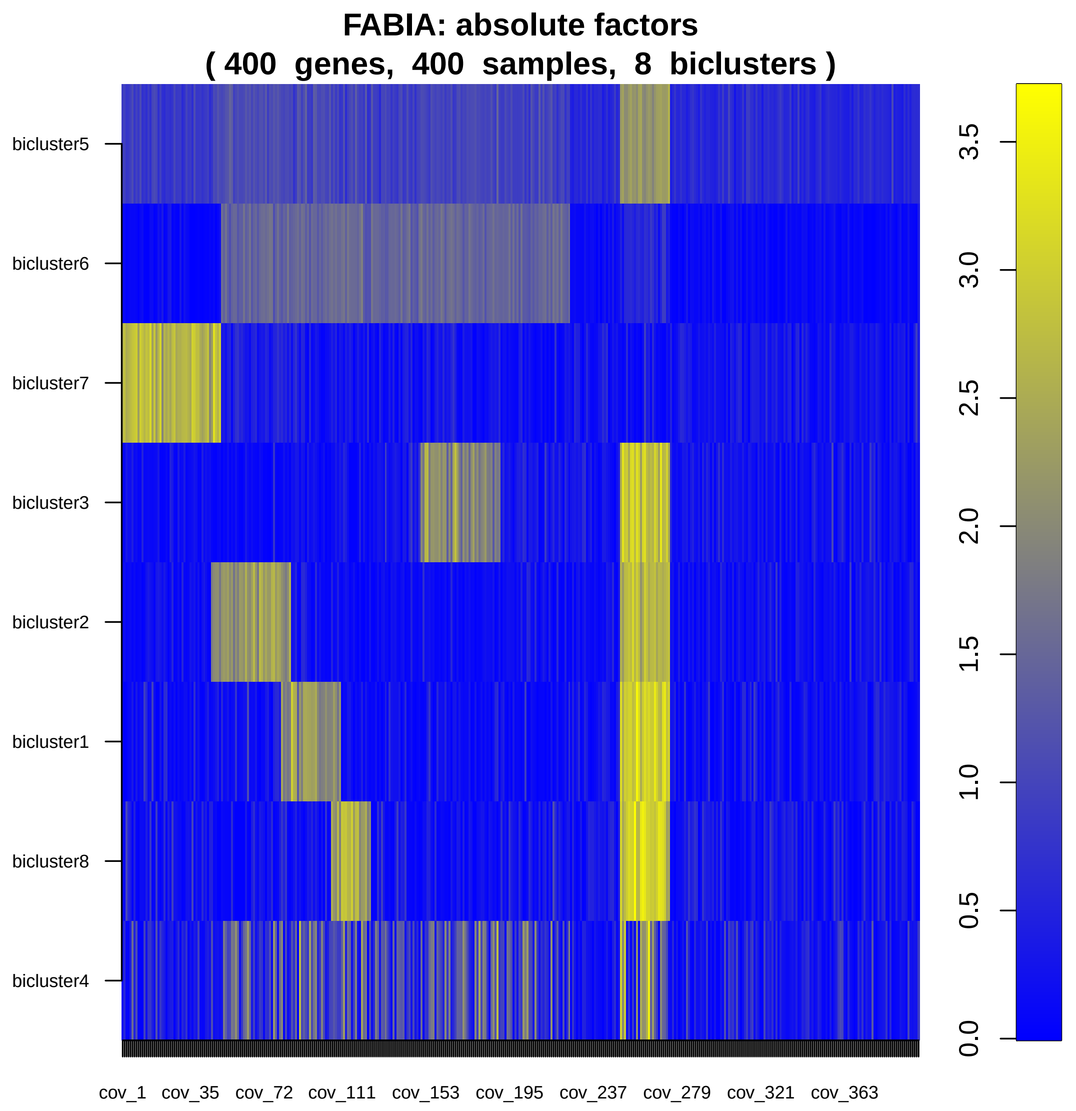

Supplementary Fig. 9. absolute loading on each factor from FABIA. Note some factors have a large numbers of phenotypes with high weights

Table 12. FABIA with parameters p=8, default alpha=0.1, cyc=1000. Alpha is the spareness loading, p is the number of clusters and cyc is the number of cycles. Accur, Sens, Spec, PPV and NPV are accuracy, sensitivity, specificity positive predictive value (precision) and negative predictive value respectively FABIA identified large clusters with many false positives

Module	JI	Cluster Size		phenotype (Col)					Gene Set (row)				
		nRow	nCol	Accur	Sens	Spec	PPV	NPV	Accur	Sens	Spec	PPV	NPV
M1	0.16	87	358	0.17	1.00	0.11	0.07	1.00	0.39	0.19	0.73	0.54	0.35
M2	0.09	12	185	0.97	1.00	0.96	0.95	1.00	0.82	0.09	0.98	0.58	0.82
M3	0.90	45	111	0.85	1.00	0.83	0.45	1.00	0.99	0.90	1.00	1.00	0.99
M4	0.80	32	79	0.90	1.00	0.89	0.51	1.00	0.98	0.80	1.00	1.00	0.98
M5	0.73	22	194	0.59	1.00	0.56	0.15	1.00	0.98	0.73	1.00	1.00	0.98
M6	1.00	20	95	0.81	1.00	0.80	0.21	1.00	1.00	1.00	1.00	1.00	1.00
M7	0.68	27	100	0.85	1.00	0.83	0.40	1.00	0.97	0.68	1.00	1.00	0.96

Table 13. Results of FABIA with parameters p=8, alpha=0.2, cyc=1000. Alpha is the spareness loading, p is the number of clusters and cyc is the number of cycles. Accur, Sens, Spec, PPV and NPV are accuracy, sensitivity, specificity positive predictive value (precision) and negative predictive value respectively FABIA identified large clusters with many false positives.

Module	JI	Cluster Size		phenotype (Col)					Gene Set (row)				
		nRow	nCol	Accur	Sens	Spec	PPV	NPV	Accur	Sens	Spec	PPV	NPV
M1	0.15	83	352	0.18	1.00	0.13	0.07	1.00	0.39	0.18	0.74	0.53	0.35
M2	0.09	10	182	0.98	1.00	0.97	0.96	1.00	0.82	0.09	0.99	0.70	0.83
M3	0.90	45	97	0.88	1.00	0.87	0.52	1.00	0.99	0.90	1.00	1.00	0.99
M4	0.80	32	105	0.84	1.00	0.82	0.38	1.00	0.98	0.80	1.00	1.00	0.98
M5	0.77	23	127	0.76	1.00	0.74	0.24	1.00	0.98	0.77	1.00	1.00	0.98
M6	1.00	20	145	0.69	1.00	0.67	0.14	1.00	1.00	1.00	1.00	1.00	1.00
M7	0.82	33	111	0.82	1.00	0.80	0.36	1.00	0.98	0.82	1.00	1.00	0.98

Table 14. Results of FABIA with parameters p=8, alpha=0.3, cyc=1000. Alpha is the spareness loading, p is the number of clusters and cyc is the number of cycles. Accur, Sens, Spec, PPV and NPV are accuracy, sensitivity, specificity positive predictive value (precision) and negative predictive value respectively. FABIA identified large clusters with many false positives.

Module	JI	Cluster Size		phenotype (Col)					Gene Set (row)				
		nRow	nCol	Accur	Sens	Spec	PPV	NPV	Accur	Sens	Spec	PPV	NPV
M1	0.27	34	93	0.83	1.00	0.82	0.27	1.00	0.44	0.12	0.97	0.88	0.40
M2	0.88	11	198	0.94	1.00	0.90	0.88	1.00	0.83	0.12	0.99	0.82	0.83
M3	0.24	49	212	0.59	1.00	0.54	0.24	1.00	1.00	0.98	1.00	1.00	1.00
M4	0.43	34	93	0.87	1.00	0.85	0.43	1.00	0.98	0.85	1.00	1.00	0.98
M5	0.21	24	143	0.72	1.00	0.69	0.21	1.00	0.98	0.80	1.00	1.00	0.98
M6	0.18	20	113	0.77	1.00	0.76	0.18	1.00	1.00	1.00	1.00	1.00	1.00
M7	0.25	39	158	0.70	1.00	0.67	0.25	1.00	1.00	0.97	1.00	1.00	1.00

Table 15. The maximum Jaccard similarity Index (JI) between M1-M7 and 200 clusters extracted from Bimax in which the row size (minr) and column (minc) size ranged 2-26 and 2-20 respectively. JI values are calculated on columns (phenotypes) only. nd indicates that no clusters were found when bimax was performed using those parameters

Module	Column	Row Size												
	size	2	4	6	8	10	12	14	16	18	20	22	24	26
M1	2	0	0	0	0	0	0	0	0	0	0	0	0	0
M1	4	0	0	0	0	0	0	0	0	0	0	0.24	0.28	0.24
M1	6	0	0	0	0	0	0	0	0	0.36	0.36	0.36	0.32	0.32
M1	8	0	0	0	0	0	0	0.36	0.4	0.36	0.4	0.36	0.36	0.32
M1	10	0	0	0	0	0	0	0.029	0.44	0.44	0.44	0.44	0.4	0.44
M1	12	0	0	0	0	0.52	0.423	0.48	0.52	0.48	0.52	0.48	nd	nd
M1	14	0	0	0	0.6	0	0.56	0.56	0.6	0.56	nd	nd	nd	nd
M1	16	0	0	0	0.025	0.051	0.68	0.64	nd	nd	nd	nd	nd	nd
M1	18	0	0	0.536	0.387	0.536	0.024	nd	nd	nd	nd	nd	nd	nd
M1	20	0	0	0.667	0	0.023	0	nd	nd	nd	nd	nd	nd	nd
M2	2	0	0	0	0	0	0	0	0	0	0	0	0	0
M2	4	0	0	0	0	0	0	0	0	0	0	0.023	0.023	0.011
M2	6	0	0	0	0	0	0	0	0	0.034	0.034	0.006	0	0
M2	8	0	0	0	0	0	0	0.011	0	0.051	0	0	0	0
M2	10	0	0	0	0	0	0.063	0.063	0	0	0	0	0	0
M2	12	0	0	0	0	0.011	0.074	0	0	0.005	0	0	nd	nd
M2	14	0	0	0	0.074	0.091	0.086	0.08	0.005	0	nd	nd	nd	nd
M2	16	0	0	0	0.114	0.12	0.109	0	nd	nd	nd	nd	nd	nd
M2	18	0.005	0.005	0.096	0.12	0.114	0.114	nd	nd	nd	nd	nd	nd	nd
M2	20	0.005	0	0.102	0.143	0.137	0.114	nd	nd	nd	nd	nd	nd	nd
M3	2	0.14	0.14	0.14	0.14	0.14	0.12	0.1	0.1	0.1	0.1	0.08	0.08	0.06
M3	4	0.16	0.16	0.16	0.16	0.14	0.12	0.12	0.12	0.12	0.1	0.08	0.08	0.08
M3	6	0.2	0.2	0.2	0.2	0.18	0.16	0.14	0.14	0.12	0	0	0	0
M3	8	0.24	0.24	0.24	0.22	0.18	0.18	0.16	0	0	0	0	0	0
M3	10	0.3	0.3	0.28	0.24	0.24	0.2	0	0	0	0	0	0	0
M3	12	0.34	0.34	0.34	0.28	0.24	0	0	0	0	0	0	nd	nd
M3	14	0.4	0.4	0.34	0.3	0	0	0	0	0	nd	nd	nd	nd
M3	16	0.4	0.46	0.38	0	0	0	0	nd	nd	nd	nd	nd	nd
M3	18	0.529	0.48	0.38	0	0	0	nd	nd	nd	nd	nd	nd	nd
M3	20	0.56	0.5	0.029	0	0	0	nd	nd	nd	nd	nd	nd	nd
M4	2	0.024	0.024	0.024	0.024	0.024	0.024	0.024	0.024	0.05	0.05	0.05	0.05	0.075
M4	4	0.023	0.023	0.023	0.023	0.023	0.023	0.048	0.048	0.048	0.1	0.1	0.1	0.1
M4	6	0.045	0.045	0.045	0.045	0.045	0.045	0.07	0.095	0.045	0.15	0	0	0
M4	8	0.043	0.043	0.043	0.043	0.043	0.091	0.067	0	0.2	0	0	0	0
M4	10	0.064	0.064	0.064	0.087	0.087	0.087	0.042	0	0	0	0	0	0
M4	12	0.083	0.083	0.083	0.083	0.061	0.106	0	0	0.02	0	0	nd	nd
M4	14	0.08	0.08	0.08	0.08	0.102	0.08	0.059	0.019	0	nd	nd	nd	nd
M4	16	0.077	0.077	0.077	0.143	0.037	0.077	0	nd	nd	nd	nd	nd	nd
M4	18	0.074	0.074	0.283	0.16	0.137	0.094	nd	nd	nd	nd	nd	nd	nd
M4	20	0.071	0.071	0.196	0.154	0.111	0.034	nd	nd	nd	nd	nd	nd	nd
M5	2	0	0	0	0	0	0	0	0	0	0	0	0	0
M5	4	0	0	0	0	0	0	0	0	0	0	0.097	0.03	0.062
M5	6	0	0	0	0	0	0	0	0	0.029	0.029	0.029	0	0
M5	8	0	0	0	0	0	0	0.027	0	0.118	0	0	0	0
M5	10	0	0	0	0	0	0.111	0.111	0	0	0	0	0	0
M5	12	0	0	0	0	0.05	0.167	0	0	0.024	0	0	nd	nd
M5	14	0	0	0	0.048	0.189	0.158	0.073	0.023	0	nd	nd	nd	nd
M5	16	0	0	0	0.095	0.15	0.15	0	nd	nd	nd	nd	nd	nd
M5	18	0	0	0.091	0.116	0.171	0.143	nd	nd	nd	nd	nd	nd	nd
M5	20	0	0	0.02	0.136	0.163	0.111	nd	nd	nd	nd	nd	nd	nd

Table 15. Continued: The maximum Jaccard similarity Index (JI) between M1-M7 and 200 clusters extracted from Bimax in which the row size (minr) and column (minc) size ranged 2-26 and 2-20 respectively. JI values are calculated on columns (phenotypes) only. nd indicates that no clusters were found when bimax was performed using those parameters.

Module	Column	Row Size												
	size	2	4	6	8	10	12	14	16	18	20	22	24	26
M6	2	0	0	0	0	0	0	0	0	0	0	0	0	0
M6	4	0	0	0	0	0	0	0	0	0	0	0.043	0.043	0.043
M6	6	0	0	0	0	0	0	0	0	0.04	0	0	0	0
M6	8	0	0	0	0	0	0	0	0	0.167	0	0	0	0
M6	10	0	0	0	0	0	0.034	0.154	0	0	0	0	0	0
M6	12	0	0	0	0	0	0.143	0	0	0	0	0	nd	nd
M6	14	0	0	0	0.03	0.133	0.062	0.03	0	0	nd	nd	nd	nd
M6	16	0	0	0	0.125	0.125	0.125	0	nd	nd	nd	nd	nd	nd
M6	18	0	0	0.027	0.147	0.147	0.118	nd	nd	nd	nd	nd	nd	nd
M6	20	0	0	0	0.111	0.081	0.026	nd	nd	nd	nd	nd	nd	nd
M7	2	0	0	0	0	0	0	0	0	0	0	0	0	0
M7	4	0	0	0	0	0	0	0	0	0	0	0.023	0.023	0.023
M7	6	0	0	0	0	0	0	0	0	0.045	0	0	0	0
M7	8	0	0	0	0	0	0	0	0	0.091	0	0	0	0
M7	10	0	0	0	0	0	0.087	0.136	0	0	0	0	0	0
M7	12	0	0	0	0	0	0.13	0	0	0	0	0	nd	nd
M7	14	0	0	0	0.149	0.125	0.102	0.08	0	0	nd	nd	nd	nd
M7	16	0	0	0	0.077	0.12	0.098	0	nd	nd	nd	nd	nd	nd
M7	18	0	0	0.055	0.137	0.115	0.094	nd	nd	nd	nd	nd	nd	nd
M7	20	0	0	0.333	0.132	0.154	0.071	nd	nd	nd	nd	nd	nd	nd

Table 16. Results of Bimax (best performance with highest JI across parameters tested in Table 15. Accur. Sens amd Spec are accuracy, sensitivity and specificity respectively. Parameters are the minr and minc listed in Table 15. 200 clusters were identified in each run and the cluster which had the highest JI to each module is listed.

Module	Best Cluster		JI	Cluster Size		phenotype (Col)					Gene Set (Row)				
	Parameters	Cluster		nRow	nCol	Accur	Sens	Spec	PPV	NPV	Accur	Sens	Spec	PPV	NPV
M1	C16, R12	55	0.68	12	17	0.98	0.68	1.00	1.00	0.98	0.41	0.05	1.00	1.00	0.39
M2	C20, R8	154	0.14	8	25	0.62	0.14	1.00	1.00	0.60	0.83	0.11	1.00	1.00	0.83
M3	C20, R2	63	0.56	3	28	0.94	0.56	1.00	1.00	0.94	0.88	0.06	1.00	1.00	0.88
M4	C18, R6	43	0.28	6	19	0.92	0.33	0.98	0.68	0.93	0.89	0.03	0.99	0.17	0.90
M5	C14, R10	78	0.19	10	14	0.93	0.23	0.98	0.50	0.94	0.90	0.00	0.97	0.00	0.92
M6	C8, R18	146	0.17	19	8	0.95	0.20	0.99	0.50	0.96	0.94	0.40	0.97	0.42	0.97
M7	C20, R6	1	0.33	6	20	0.93	0.38	0.99	0.75	0.93	0.89	0.03	0.99	0.17	0.90

Table 17. Results of Bimax with parameters R22 and C4, which had highest JI sum over the 7 modules. JI between columns and rows are provided. Accur. Sens amd Spec are accuracy, sensitivity and specificity respectively. 200 clusters were identified in each run and the cluster which had the highest JI to each module is listed.

Module	Best Cluster	JI		Cluster Size		phenotype (Col)					Gene Set (Row)				
		(col)	(row)	nRow	nCol	Accur	Sens	Spec	PPV	NPV	Accur	Sens	Spec	PPV	NPV
M1	168	0.24	0.30	41	6	0.95	0.24	1.00	1.00	0.95	0.48	0.16	1.00	1.00	0.42
M2	101	0.02	0.30	22	4	0.57	0.02	1.00	1.00	0.57	0.86	0.28	1.00	0.95	0.86
M3	1	0.08	0.50	24	4	0.88	0.08	1.00	1.00	0.88	0.94	0.48	1.00	1.00	0.93
M4	33	0.10	0.37	26	4	0.91	0.10	1.00	1.00	0.91	0.88	0.25	0.96	0.38	0.92
M5	110	0.10	0.40	23	4	0.93	0.10	1.00	0.75	0.93	0.92	0.37	0.97	0.48	0.95
M6	109	0.04	0.14	22	4	0.94	0.05	0.99	0.25	0.95	0.90	0.00	0.94	0.00	0.95
M7	101	0.02	0.17	22	4	0.90	0.02	0.99	0.25	0.90	0.84	0.00	0.94	0.00	0.89

5 ANALYSIS OF BREAST CANCER DATA:

5.1 Discovery of new modules - analysis of GSVA data

GSVA was performed using default parameters on each dataset and the distribution of results were explored to determine a threshold at which the data would be discretized. We selected a threshold of ± 0.3 as it would yield a matrix with approximately 10% of associations. A histogram should the statistics from each analysis is given below

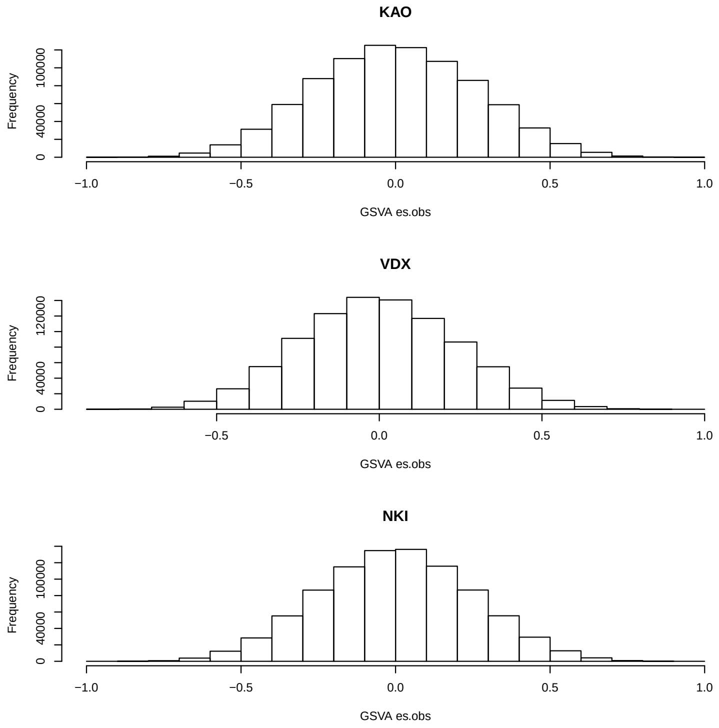

Supplementary Fig. 10. Distributions of results of GSVA analysis of each of the three breast cancer datasets (KAO, VDX, NKI)

Results in the Table 18 summarize the 13 modules in results of GSVA analysis of 3 breast cancer datasets (KAO, NKI and VDX). Overal Survival (O.Surv.) and Distant Metastasis Free surival analysis was performed using a cox regression model (coxph in R) and pvalue were FDR corrected for multiple testing. Cells highlights in red indicate modules significantly associated with poor prognosis whereas those in green are associated with a favorable outcome. Cells highlighted in yellow and blue respresented modules that were over or under represented in a breast cancer molecular subtypes respectively, the analysis of which was performed using a Fisher extact test and resulting p-value were corrected for multiple testing using a FDR correction.

Table 18. Modules (n=13) discovered by iBBiG in analysis of results of GSVA of gene expression profiles from three breast cancer study. Colors highlight modules significantly enriched in breast cancer molecular subtype by Fisher exact test (greater, yellow; less, blue) or prognostic of good (green) or poor (red) overall (OS) or distant metastases free survival (DMFS) ether within a molecular subtypes or across all cancers

	Gene Sets		Phenotypes			Molecular Subtype				Prognostic in Molecular Subtypes				All Subtypes	
	N	Description	N	Description	KAO/VDX/NKI	LumA	LumB	Her2	Basal	Lum A	Lum B	Her2	Basal	OS	DMFS
M1	206	Cell Cycle check-point genes, ESR1	215	Luminal A, Good prognosis	78/62/75	0.000	0.000	0.000	0.000	dmfs				0.000	0.000
M2	265	Proliferation up, ES up (Ben porath signatures), IFN	163	Basal-Like, Poor prognosis	59/54/50	0.000	0.000	NS	0.000		OS, dmfs			0.001	0.050
M3	263	ESR1 genes,Immune	127	Luminal A or B	58/36/33	0.002	0.003	0.011	0.000				dmfs	NS	NS
M4	209	Inflammatory response	161	Basal-like or HER2	73/47/41	0.000	0.000	0.000	0.000					NS	NS
M5	348	Stromal	63	Overlap M1,M4	33/11/19	NS	0.001	NS	NS					NS	NS
M6	294	Stromal	27	Luminal B (subset of M2, M3)	14/1/12	NS	0.029	NS	NS					NS	NS
M7	412	Immune response (maybe B -cells)	23	Overlap with M4	18/2/3	NS	NS	NS	NS					NS	NS
M8	104	Immune response (IFN)	115	Luminal A (some B) No overlap with M1	37/30/48	0.000	NS	0.004	0.000		dmfs			NS	NS
M9	447	proliferation up	7	Overlaps with M3	3/3/1	NS	NS	NS	NS					NS	NS
M10	177	**Metastatic**	35	Poor prognosis, no overlaps	13/9/13	NS	NS	NS	NS	dmfs		dmfs		NS	0.002
M11	383	Stromal	17	Poor prognosis, overlaps M4	13/2/2	NS	NS	NS	NS				dmfs	NS	0.080
M12	529	AKT, HIF	5	Overlap M2	3/2/0	NS	NS	NS	NS					NS	NS
M13	271	metabolism	19	Luminal A, overlaps M1	9/1/9	0.000	NS	NS	0.044					NS	NS

5.2 Discovery of new modules - M1

The first module identified contained 215 phenotypes which were enriched in estrogen receptor positive low grade luminal A breast cancer. Expression of these gene sets (n=206) was associated with a better outcome in breast cancer patients

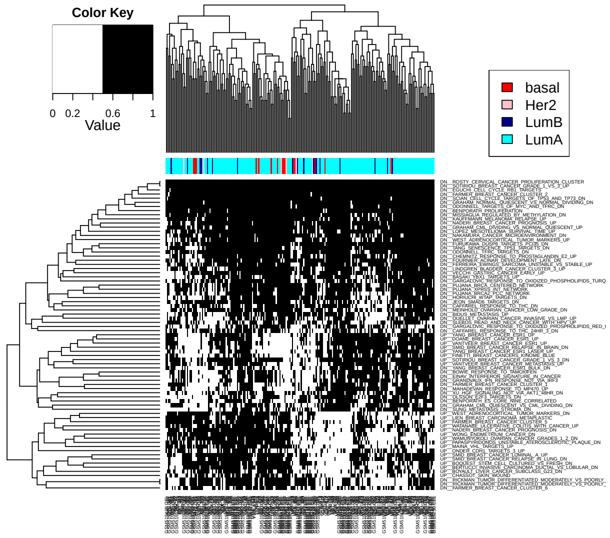

Supplementary Fig. 11. Heatmap showing genesset and phenotypes which characterize module M11

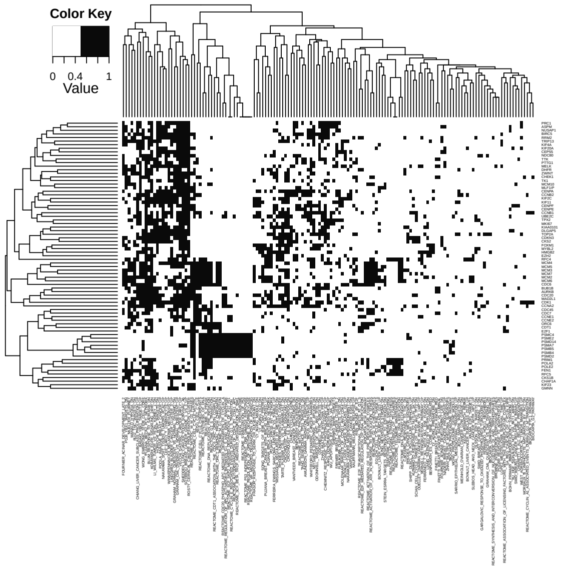

Supplementary Fig. 12. Heatmap showing genes which were significantly differentially requlated (limma lmfit/eBayes p¡0.001) and are members of gene sets in M1

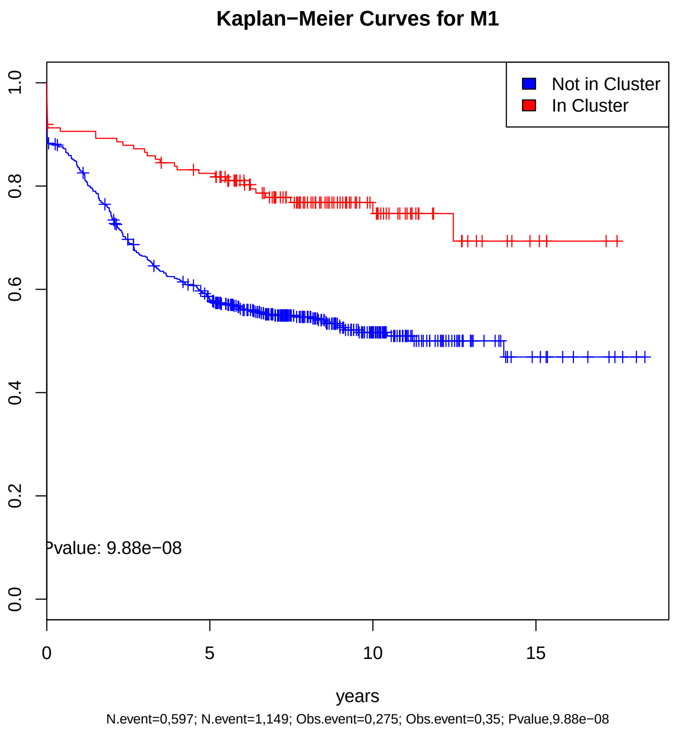

Supplementary Fig. 13. KM curve showing the ability of M1 to distinguish breast cancer patients with better distant metastases free survival

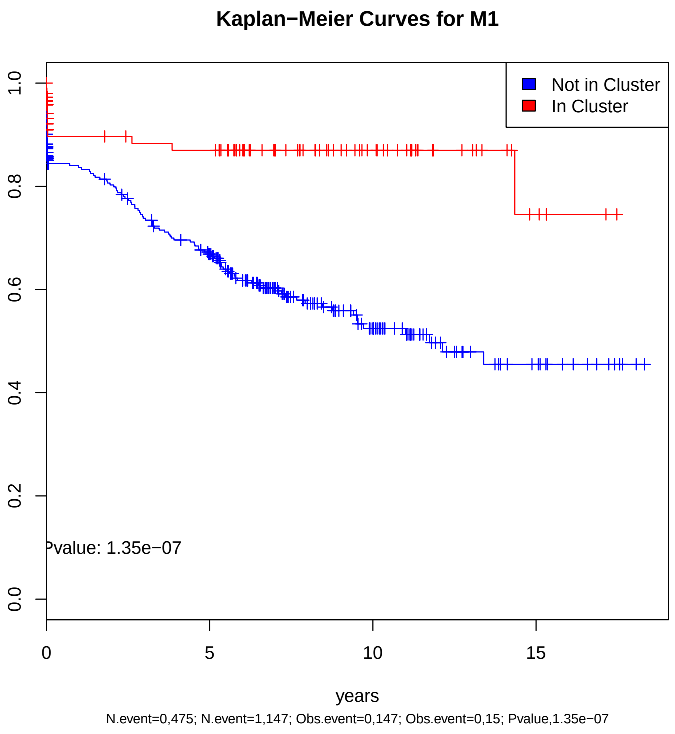

Supplementary Fig. 14. KM curve showing the ability of M1 to distinguish breast cancer patients with better overall survival

5.3 Discovery of new modules - M2

The second module identified contained 163 phenotypes which were enriched in estrogen receptor negative high grade basal-like breast cancer. Expression of these gene sets (n=265) was associated with a poorer outcome in breast cancer patients

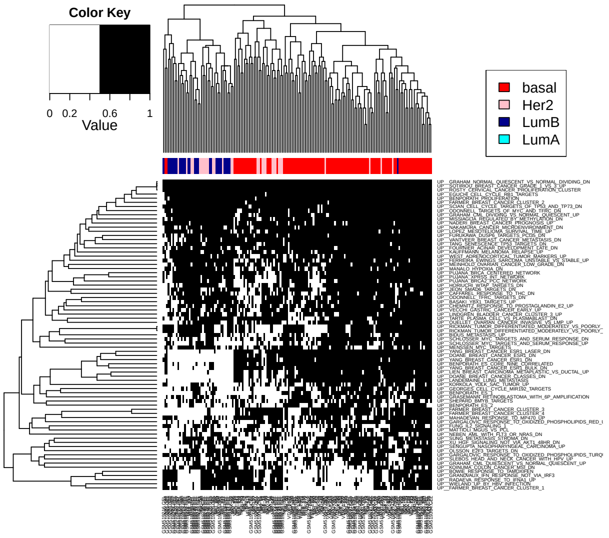

Supplementary Fig. 15. Heatmap showing genesset and phenotypes which characterize module M2

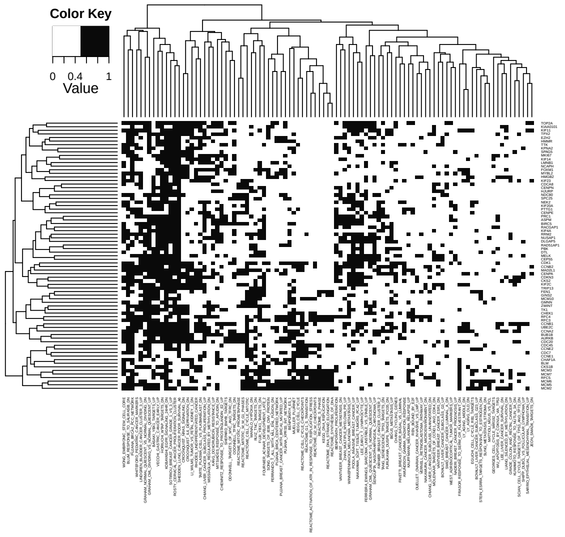

Supplementary Fig. 16. Heatmap showing genes which were significantly differentially requlated (limma lmfit/eBayes p¡0.001) and are members of gene sets in M2

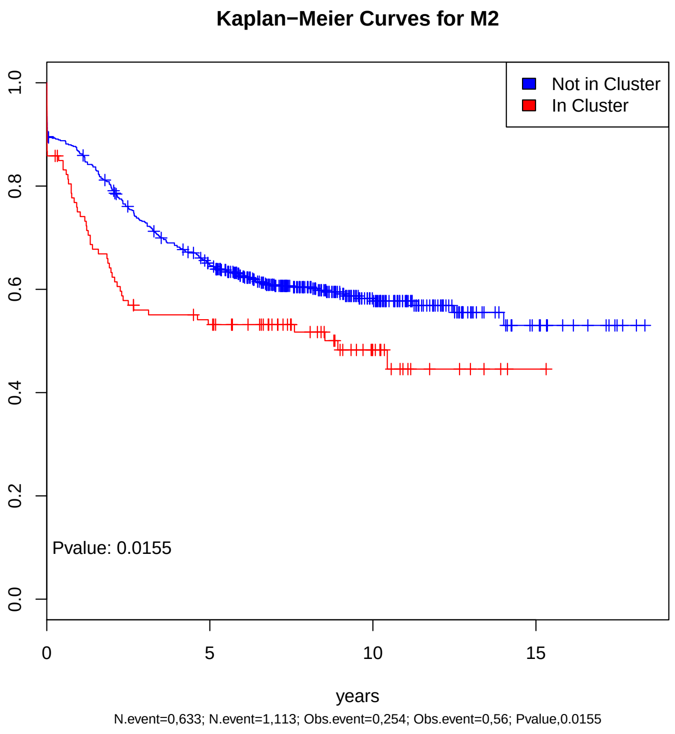

Supplementary Fig. 17. KM curve showing the ability of M2 to distinguish breast cancer patients with better distant metastases free survival

5.4 Discovery of new modules - M10

The second module identified contained 163 phenotypes which were enriched in estrogen receptor negative high grade basal-like breast cancer. Expression of these gene sets (n=265) was associated with a poorer outcome in breast cancer patients

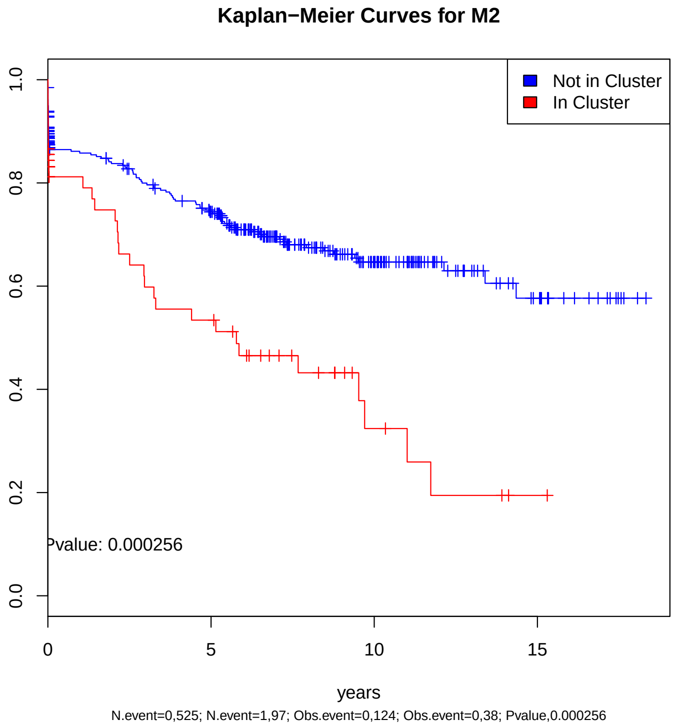

Supplementary Fig. 18. KM curve showing the ability of M2 to distinguish breast cancer patients with better overall survival

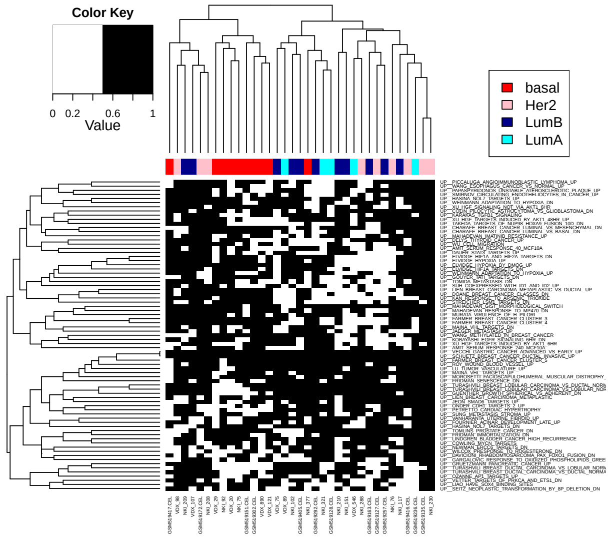

Supplementary Fig. 19. Heatmap showing genesset and phenotypes which characterize module 10

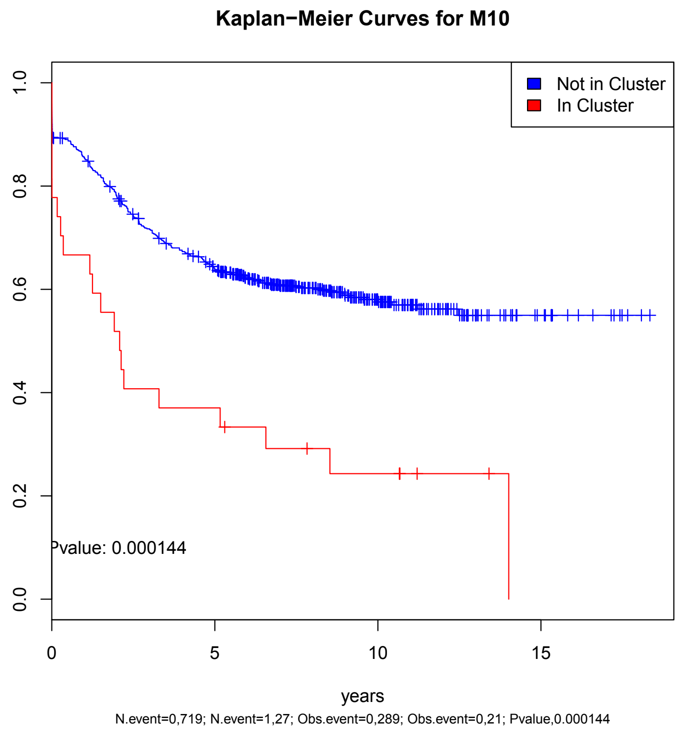

Supplementary Fig. 20. KM curve showing the ability of M10 to distinguish breast cancer patients with and without distant metastases free survival

Table 19. There were 177 gene sets in M10 and the 20 gene sets with highest scores are provided. Each of these were upregulated in M10

Gene Sets	Score
REN_ALVEOLAR_RHABDOMYOSARCOMA_DN	28.732498
CROMER_TUMORIGENESIS_UP	27.914161
VECCHI_GASTRIC_CANCER_ADVANCED_VS_EARLY_UP	27.63875
ROZANOV_MMP14_TARGETS_SUBSET	23.679678
SCHUETZ_BREAST_CANCER_DUCTAL_INVASIVE_UP	23.653064
FARMER_BREAST_CANCER_CLUSTER_5	23.512512
MAHADEVAN_GIST_MORPHOLOGICAL_SWITCH	21.209128
TURASHVILI_BREAST_LOBULAR_CARCINOMA_VS_DUCTAL_NORMAL_UP	21.208569
ROY_WOUND_BLOOD_VESSEL_UP	21.070373
TURASHVILI_BREAST_LOBULAR_CARCINOMA_VS_LOBULAR_NORMAL_DN	20.669667
LU_TUMOR_VASCULATURE_UP	20.329544
MISHRA_CARCINOMA_ASSOCIATED_FIBROBLAST_UP	20.138516
HASINA_NOL7_TARGETS_UP	19.081524
LU_TUMOR_ANGIOGENESIS_UP	19.072392
BRUECKNER_TARGETS_OF_MIRLET7A3_DN	18.460937
ALONSO_METASTASIS_NEURAL_UP	18.276682
MAINA_VHL_TARGETS_UP	17.989316
VERRECCHIA_RESPONSE_TO_TGFB1_C1	17.982409
CROONQUIST_STROMAL_STIMULATION_UP	17.914741

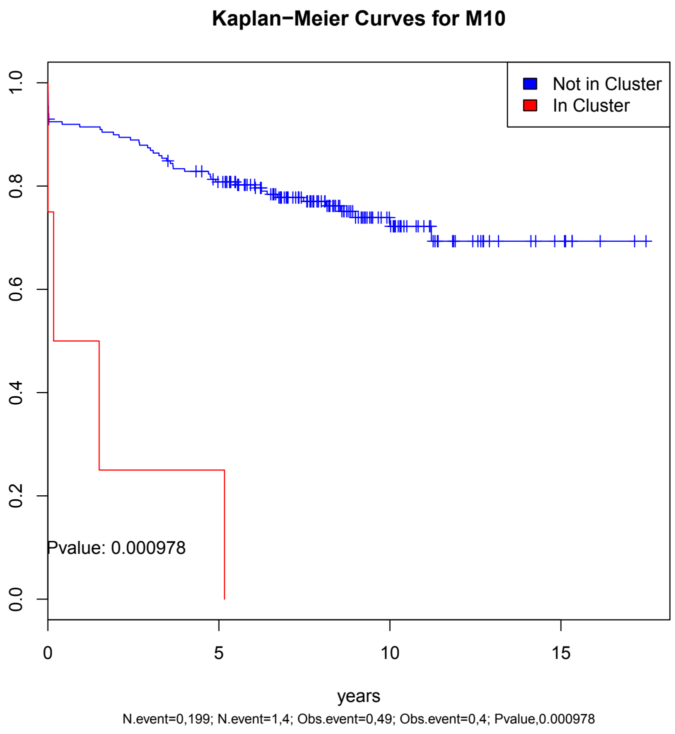

Supplementary Fig. 21. KM curve showing the ability of M10 to distinguish Luminal A molecular subtype breast cancer patients with and without distant metastases free survival

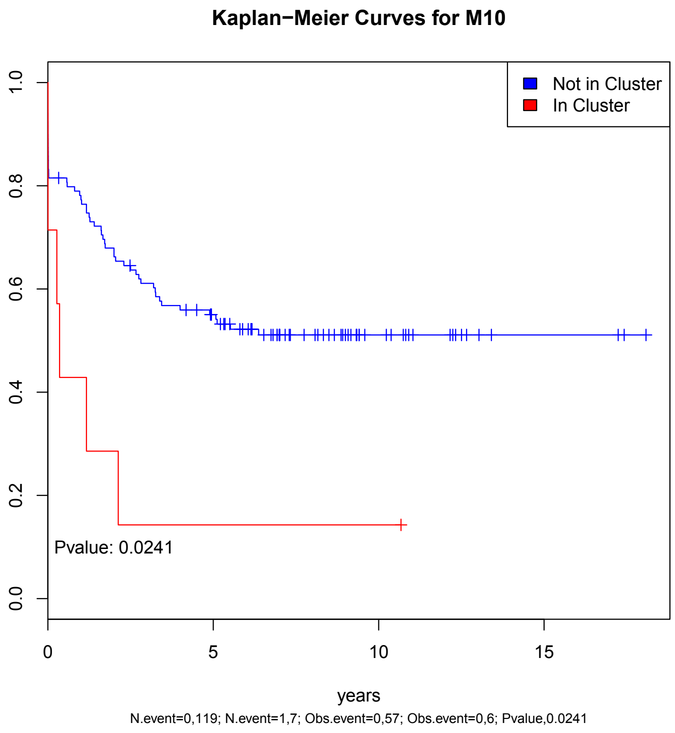

Supplementary Fig. 22. KM curve showing the ability of M10 to distinguish ERBB2 molecular subtype breast cancer patients with and without distant metastases free survival

5.5 Discovery of modules associated with known clinical covariates in breast cancer

Results of bi-cluster analysis with iBBiG on the breast cancer dataset with 50 modules

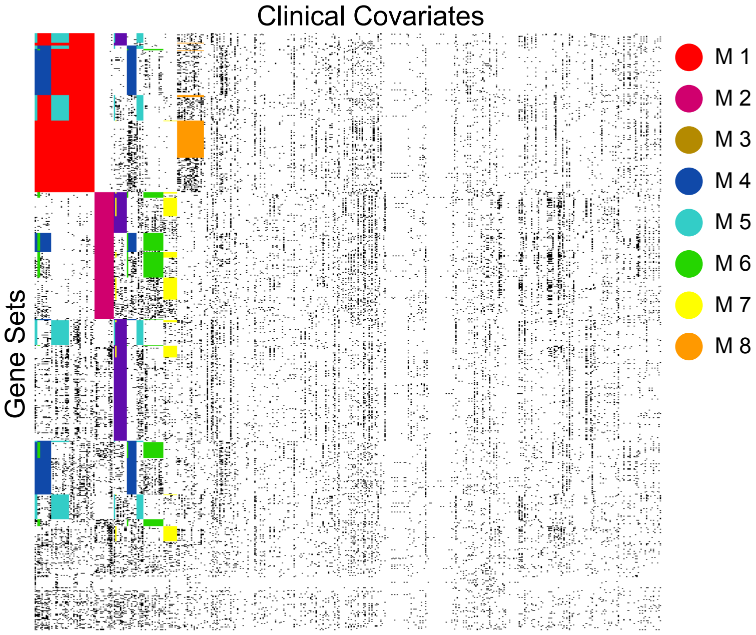

Supplementary Fig. 23. Meta-GSA using iBBiG bi-clustering associations between 2853 gene sets and 448 clinical covariates resulting from the gene set enrichment analysis of 22 breast cancer datasets. The first eight modules (M1-M8) are shown. The covariates and the gene sets are ordered according to module membership (Detailed description in Supplement). The fitness score and size of modules are provided in Supplement Figure S5 and a more detailed view of the gene sets and covariates that make up module (M4) are provided in supplementary Figure S6.

Table 20. Summary of the eight resulting breast cancer modules B1-B8 resulting from iBBiG cluster discovery in results of GSEAlm gene set enrichment analysis of genes that were differentially expressed between pairwise tests of 10446 clinical covariates

	Number	Module Size			
	Datasets	Pairwise Tests	Gene Sets	Pairwise Tests	Gene Sets
B1	14	43	247	High grade, basal / luminal B, mutant p53, ER-, PR-, immortal, relapse, cell line	DNA replication, cell cycle, mitosis, M-phase, spindle, DNA metabolic process and apoptotic mitochondrial changes
B2	7	9	262	High grade, untreated, resistant, immortal	Wound healing, coagulation, response to light stimulus, cell-cell signaling, excretion, ion channel activity, transmembrane receptor activity and plasma membrane
B3	6	14	270	Low grade, wild-type p53, normal like breast tissue	Developmental maturation, enzyme linked receptor protein signaling, cell maturation, basolateral plasma membrane, basal lamina, negative regulation of cell differentiation and extracellular matrix
B4	11	21	75	High grade, basal / luminal B, mutant p53, ER-, PR-, no metastasis	Regulation if immune system process, IL17 pathway, protein kinase cascade, T-cell receptor signaling, lymphocyte activation and chemokine activation
B5	8	19	89	Cell line, luminal, low stage, metastatic	DNA directed RNA polymerase, endoplasmic reticulum, protein catabolic process, RNA splicing, ubiquitin protein ligase activity, cellular protein catabolic process, secondary metabolic process and citrate cycle
B6	9	17	74	Tamoxifen treated, luminal, ER+, PR+	Synaptic vesicle, secretion by cell, vesicle mediated transport, intrinsic to Golgi membrane, sphingoid metabolic process, Golgi vesicle transport and Golgi stack
B7	4	11	115	No relapse, no subtype, high grade	Insulin like growth factor receptor binding, extracellular matrix, myoblast differentiation, actin binding, muscle cell differentiation, focal adhesion, muscle development and cell matrix junction
B8	10	19	62	High stage, ER-, metastatic, basal, ductal	Cell cycle process, chromosome segregation, mitosis, cell cycle checkpoint, interphase and, condensed chromosome

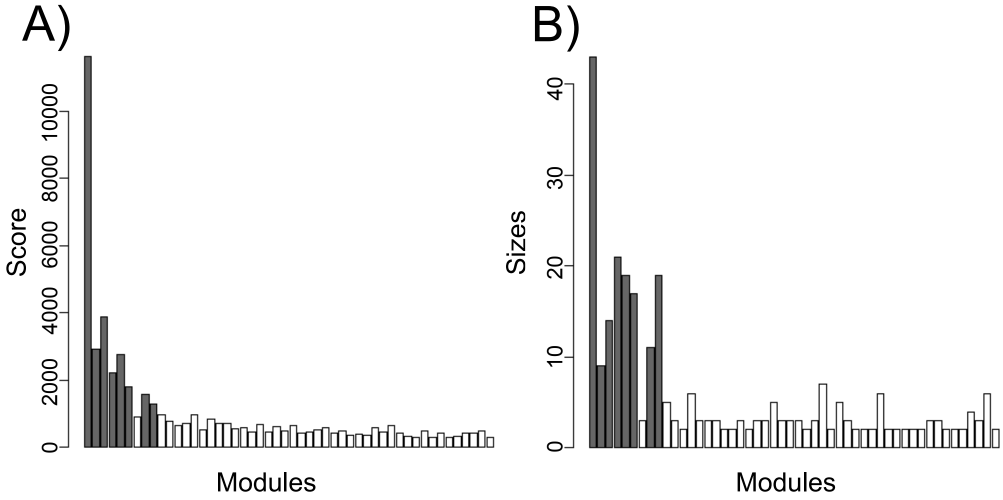

Supplementary Fig. 24. Results of bi-cluster analysis with iBBiG on the breast cancer dataset with 50 modules; A) histogram of the size. Selected modules are shown in gray; modules that we not used are shown in white. B) Fitness scores of the first 50 modules.

The B4 module also included seven gene signatures from published articles that were indexed by GeneSigDB (Culhane *et al.*, 2009) from studies of leukemia ($n = 4$), stomach, colon and 95 gene signatures of STAT1 immune signaling in breast cancer (Supplemental Figure 26). 15 genes were represented in at least two of the seven gene signatures. These included key immune and T-cell regulatory gene CCL5, which was prognostic of better outcome in Basal-like and ERBB2+ breast cancer in a meta-analysis of six publicly available datasets (Supplemental Figure 26).

Table 21. Examples of the most common pairwise tests within our breast cancer datasets. The first column indicates the clinical covariate, the second the different classes that are contained in the covariate and the third the different pairwise tests that were applied.

Clinical Covariate	Class	Pairwise-Test
ER	{pos,neg}	ER pos vs. ER neg
PR	{pos,neg}	PR pos vs. PR neg
LN	{pos,neg}	LN pos vs. LN neg
HER2	{pos,neg}	HER2 pos vs HER2 neg
p53	{pos,neg}	p53 mutant vs. p53 wildtype
Stage	{1, 2, 3, 4}	1 vs.2, 1 vs. 3, 1 vs. 4, 2 vs. 3, 2 vs. 4, 3 vs. 4
grade	{1, 2, 3}	1 vs. 3, 2 vs. 3, 1 vs. 3
relapse	{yes,no}	relapse vs. no relapse
rfs	{yes,no}	yes vs. no
dfms	{yes,no}	yes vs. no
tumor subtype	{Normal like, Luminal A, Luminal B, ERBB2, Basal}	normal-lumA, normal-lumB, normal-ERBB2, normal-Basal, lumA-lumB, lumA-ERBB2, lumB-Basal,lumB-ERBB2, lumB-Basal, ERBB2-Basal
disease state	{Tumor, Normal}	Tumor vs. Normal
metastasis	{yes,no}	metastasis vs. no metastasis
tamoxifen treatment	{yes,no}	tamoxifen treatment vs. no treatment
cell type	{lobular, ductal}	lobular vs. ductal
tissue type	{tumor epithelium, stroma}	tumor epithelium vs. stroma

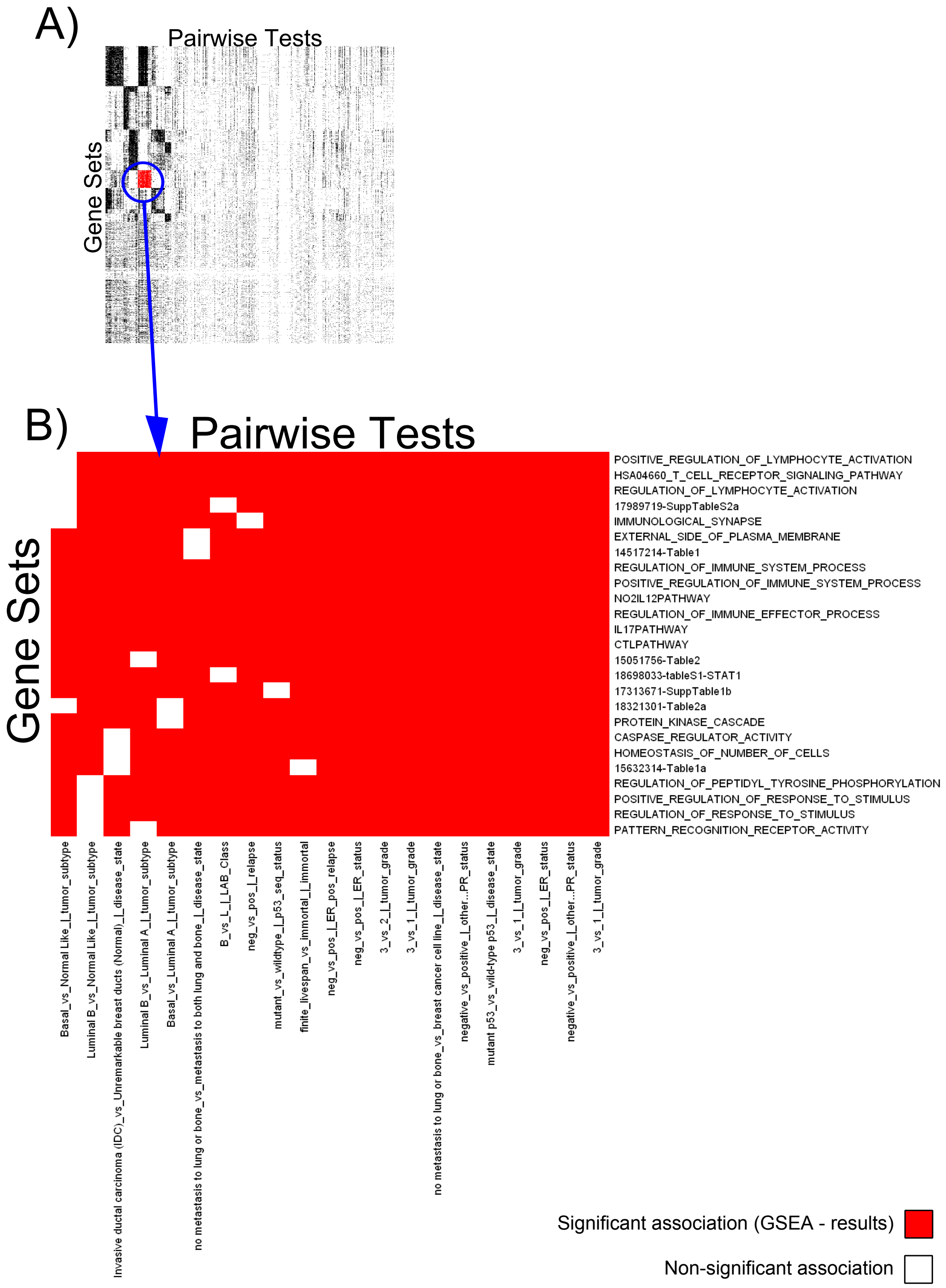

Supplementary Fig. 25. iBBiG clustering results on the breast cancer dataset. A) All associations between phenotypes and gene sets. Module 4 (B4) is highlighted in red. B) Detailed view of B4, showing all pair-wise tests in pairwise tests (n=21) and the top ranking gene sets (n=25). M4 shows the link between high grade, hormonal negative, basal-like breast cancer and an elevated immune response.

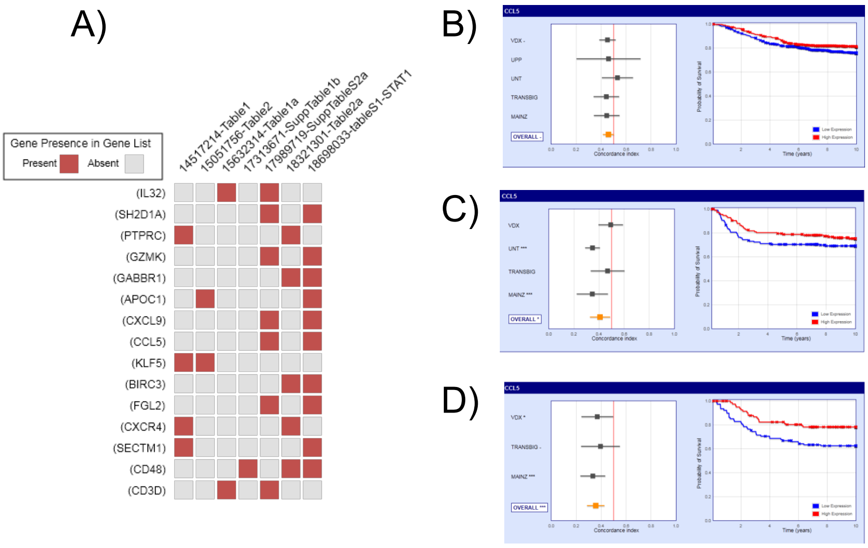

Supplementary Fig. 26. Genes that were present in at least two of the GeneSigDB gene signatures that were significant in Breast Cancer module B4 are shown in A) heatmap showing the presence (red) or absence (gray) of genes in B4 GeneSigDB gene signatures. Most of these genes were associated with good prognosis and we shown results of survival analysis of CCL5 in six breast cancer datasets in breast cancer molecular subtypes B) Luminal C) Basal-like and D) ERBB2+ which has an overall c-index of 0.458, 0.408 and 0.356 respectively.

5.6 Analysis of NHGRI GWAS catalog

Finally,iBBiG was applied to the extremely sparse NHGRI GWAS catalog to demonstrate that the method is capable of detecting small clusters in addition to larger clusters

iBBiG was applied to discretized data from the NHGRI genome wide association study (GWAS) catalog (accessed 3/7/2012), which contained data on 619 diseases (traits) and 7463 genes (Hindorff *et al.*, 2009) extracted from 1186 publications, in which only 0.3% of data had an association (i.e. one or more publications had reported a significant ($p < 1.0x10e - 5$) single nucleotide polymorphism (SNP) association between the gene and the trait).

We ran iBBiG to detect an excess of clusters, but only a few clusters were detected in this data when we applied iBBiG to the matrix of genes x traits. This matrix is extremely spare and few small clusters are expected. Note no modules was identified that had a positive weighted score, indicating that iBBiG had low confidence in these modules. Therefore we ran iBBiG 100 times, and present average analysis of these runs. Only gene and traits that occured is at least of 65% of runs are presented.

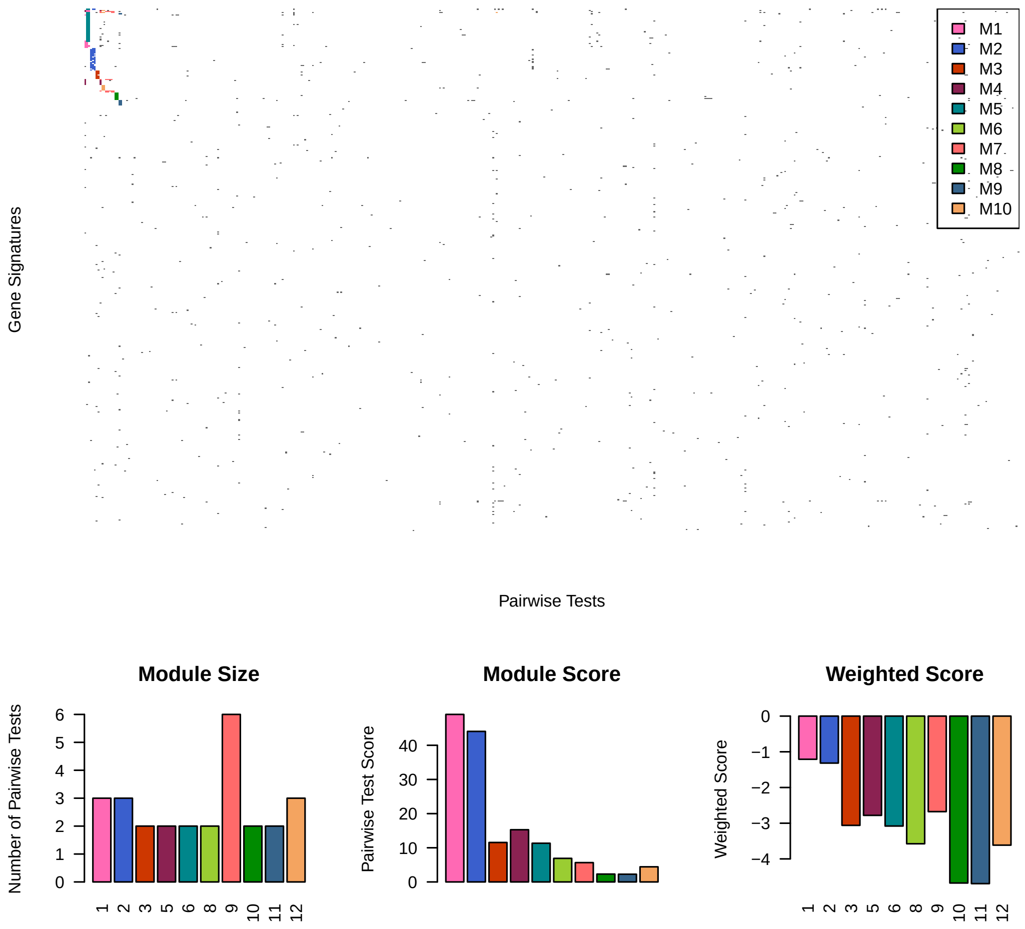

Supplementary Fig. 27. iBBiG results of analysis of the extremely sparse GWAS catalag data.

Table 22. Size (nrow, ncol), Genes identifiers (hgnc gene symbols) and Traits of Modules identified by iBBiG in the GWAS catalog data

Module 1
37 3

		Cholesterol, total	HDL cholesterol	LDL cholesterol
10452	TOMM40	1	1	1
124989	C17orf57	1	0	1
158219	TTC39B	1	1	0
19	ABCA1	1	1	0
1952	CELSR2	1	0	1
2444	FRK	1	0	1
255738	PCSK9	1	0	1
2646	GCKR	1	0	1
29116	MYLIP	1	0	1
29881	NPC1L1	1	0	1
3077	HFE	1	0	1
3122	HLA-DRA	1	0	1
3156	HMGCR	1	0	1
3172	HNF4A	1	1	0
3250	HPR	1	0	1
338	APOB	1	1	1
341	APOC1	1	1	1
3949	LDLR	1	0	1
3992	FADS1	1	1	1
5339	PLEC	1	0	1
55219	TMEM57	1	0	1
57678	GPAM	1	0	1
57794	SUGP1	1	0	1
64240;64241	ABCG5;ABCG8	1	0	1
64241	ABCG8	1	0	1
64757	MOSC1	1	0	1
6484	ST3GAL4	1	0	1
6580	SLC22A1	1	0	1
6927	HNF1A	1	0	1
7150	TOP1	1	0	1
7227	TRPS1	1	1	0
85440	DOCK7	1	0	1
8701	DNAH11	1	0	1
8882	ZNF259	1	1	1
91937	TIMD4	1	0	1
94039	ZNF101	1	0	1
9415	FADS2	1	1	1

Module 2
22 3

		Blood pressure	Diastolic blood pressure	Systolic blood pressure
10019	SH2B3	0	1	1
143872	ARHGAP42	1	1	1
144100	PLEKHA7	1	1	1
1445	CSK	1	1	1
2122	MECOM	1	1	1
219621	C10orf107	1	1	0
2242	FES	1	1	1
22834	ZNF652	1	1	0
22978	NT5C2	1	0	1
2982	GUCY1A3	1	1	1

3077	HFE	0	1	1
4343	MOV10	1	1	1
4524	MTHFR	1	0	1
490	ATP2B1	0	1	1
51196	PLCE1	1	1	1
54897	CASZ1	1	0	1
54986	ULK4	1	1	0
6311	ATXN2	1	1	0
64116	SLC39A8	1	1	1
783	CACNB2	1	1	1
7917	BAT3	0	1	1
9570	GOSR2	1	0	1

Module 3
8 2

		Crohn's disease	Ulcerative colitis
149233	IL23R	1	1
150962	PUS10	1	1
159296	NKX2-3	1	1
4485	MST1	1	1
60468	BACH2	1	0
64170	CARD9	1	1
8927	BSN	1	1
9966	TNFSF15	1	1

Module 4
8 2

		HDL cholesterol	Triglycerides
1071	CETP	1	1
144348;100533183	ZNF664	1	1
2590	GALNT2	1	1
26608	TBL2	1	1
3992	FADS1	1	1
4023	LPL	1	1
63935	PCIF1	1	1
8882	ZNF259	1	1

Module 5
29 2

		Cholesterol, total	LDL cholesterol
10452	TOMM40	1	1
124989	C17orf57	1	1
1952	CELSR2	1	1
2444	FRK	1	1
255738	PCSK9	1	1
2646	GCKR	1	1
29116	MYLIP	1	1
29881	NPC1L1	1	1
3077	HFE	1	1
3122	HLA-DRA	1	1
3156	HMGCR	1	1
3250	HPR	1	1
3949	LDLR	1	1
5339	PLEC	1	1
55219	TMEM57	1	1
57678	GPAM	1	1
57794	SUGP1	1	1

64240;64241	ABCG5;ABCG8	1	1
64241	ABCG8	1	1
64757	MOSC1	1	1
6484	ST3GAL4	1	1
6580	SLC22A1	1	1
6927	HNF1A	1	1
7150	TOP1	1	1
85440	DOCK7	1	1
8701	DNAH11	1	1
91937	TIMD4	1	1
94039	ZNF101	1	1
9415	FADS2	1	1

Module 6
7 2

		Metabolic traits	Serum metabolites
2646	GCKR	1	1
3818	KLKB1	1	1
3992	FADS1	1	1
5481	PPID	1	1
57818	G6PC2	1	1
5980	REV3L	1	1
7840	ALMS1	1	1

Module 7
5 6

		HDL Cholesterol - Triglycerides (HDLC-TG)	Metabolic syndrome	Metabolic sy
2646	GCKR	0	0	0
4023	LPL	1	1	1
84811	BUD13	1	1	1
8882	ZNF259	1	1	1
8882;116519	ZNF259;APOA5	1	1	0

	Triglycerides	Triglycerides-Blood Pressure (TG-BP)	Waist Circumference - Triglycerides (WC
2646	1	1	1
4023	1	1	0
84811	1	1	1
8882	1	1	1
8882;116519	0	1	1

Module 8
7 2

		Body mass inde	Weight
100289003	LOC100289003	1	1
25970	SH2B1	1	1
4026	LPP	1	1
497258	BDNFOS	1	1
627	BDNF	1	1
79068	FTO	1	1
89866	SEC16B	1	1

Module 9
6 2

		Coronary heart disease	Myocardial infarction (early onset)
100048912	CDKN2BAS	1	1
1952	CELSR2	1	1
221692	PHACTR1	1	1
375056	MIA3	1	1
55759	WDR12	1	1

```
6597      SMARCA4  1                    1


Module 10
10  3
              Hematological and biochemical traits Metabolic traits Serum metabolites
2646  GCKR    1                                  1              1
28    ABO     1                                  1              0
3818  KLKB1   0                                  1              1
3992  FADS1   0                                  1              1
5481  PPID    0                                  1              1
57818 G6PC2   0                                  1              1
5980  REV3L   0                                  1              1
6205  RPS11   1                                  0              1
7840  ALMS1   0                                  1              1
8170  SLC14A2 1                                  0              1
```

REFERENCES

Culhane, A. C. *et al.* (2009). GeneSigDBa curated database of gene expression signatures. *Nucleic Acids Research*, **38**(Database), D716D725.

Hindorff, L. A. *et al.* (2009). Potential etiologic and functional implications of genome-wide association loci for human diseases and traits. *Proceedings of the National Academy of Sciences of the United States of America*, **106**(23), 9362–9367. PMID: 19474294.
